# First insight into metal binding proteins from the *de novo* transcriptome of acanthocephalan parasite *Dentitruncus truttae*

**DOI:** 10.1038/s41598-025-11623-5

**Published:** 2025-07-18

**Authors:** Sara Šariri, Irena Vardić Smrzlić, Tatjana Mijošek Pavin, Vlatka Filipović Marijić

**Affiliations:** Ruđer Bošković Institute, Bijenička cesta 54, Zagreb, 10000 Croatia

**Keywords:** RNA sequencing, *de novo* transcriptome, Fish parasite, MeBiPred, Metal-binding proteins, Genetics, Molecular biology

## Abstract

Acanthocephala are parasites increasingly used as bioindicators of environmental quality due to their ability to effectively accumulate metals. However, the mechanisms of metal homeostasis in them remain unclear as there has been only one genomic study on Acanthocephala species (*Pomphorhynchus laevis*). In the present study, the transcriptome of the freshwater acanthocephalan *Dentitruncus truttae* was assembled *de novo* and analyzed for orthologs and metal-binding proteins (MBPs), which were compared between two acanthocephalans and taxa related to them phylogenetically or by lifestyle. MBPs were characterized using the PFAM database and the MeBiPred software. Orthology analysis revealed that 75% of orthogroups were species-specific, with *D. truttae* sharing most orthologues (21% of non-species-specific) with *P. laevis*. The proteome of *D. truttae* consisted of 14.5% MBPs, predominantly zinc-binding proteins such as zinc finger proteins. Phylogenetic analysis of metalloproteases (zinc-binding), iron-sulphur protein group (iron-binding) and nickel-binding ureases/hydrogenases showed that the analyzed sequences are fairly conserved across all taxonomic groups, with a particularly high conservation in Acanthocephala and Rotifera. Protein sequences that could not be described using the PFAM database were grouped into three clusters characterized by a high preference for binding zinc and copper. This study provides the first transcriptomic insights into *D. truttae* and its MBPs, contributing to future research of the molecular mechanisms underlying metal accumulation in acanthocephalans. These findings may highlight their potential as bioindicators, reveal mechanisms of tolerance to toxic metals, and improve our understanding of their ecological adaptations and roles in aquatic ecosystems.

**Supplementary Information:**

The online version contains supplementary material available at 10.1038/s41598-025-11623-5.

## Introduction

Acanthocephala, also known as thorny-headed worms, are a monophyletic group of obligate endoparasites found worldwide in arthropods and vertebrates, primarily fishes. They are recognized as model species for various research areas including host–parasite interactions, evolution of parasitic life cycles, environmental parasitology, taxonomy, phylogeography and behavioral ecology^1^. *Dentitruncus truttae* Šinžar, 1955, an acanthocephalan freshwater species, is found in restricted areas of Bosnia and Herzegovina, Italy, and Croatia. It belongs to the family Leptorhynchoididae Witenberg, 1932 (Palaeacanthocephala) and is the only member of the genus *Dentitruncus*. This species is characterized by 18 longitudinal rows of hooks on its proboscis, with 18 hooks per row (occasionally 19–20). High infestation intensity of *D. truttae* has been reported in its native habitat, the Krka river in Croatia^2–4^.

The definitive host of *D. truttae* is primarily the brown trout (*Salmo trutta* Linnaeus, 1758), while intermediate hosts include crustaceans from the genera *Gammarus* and *Echinogammarus*^2,5^. *D. truttae*, like other acanthocephalans, effectively accumulates metals, especially toxic metals, making it a bioindicator of anthropogenic metal pollution in aquatic environments^4,6,7^. In addition, infection with these parasites has been associated with reduced accumulation of toxic metals in the fish host^6,7^. Thus, *D. truttae* is significant not only as a pathogen of salmonid fish commonly consumed by humans, but also as an evolutionarily intriguing species of acanthocephalan, and as a potential bioindicator of metal pollution in freshwater ecosystems.

Transcriptome analysis enables genome-wide identification of novel genes and protein families, including metal-binding proteins (MBPs)^8–16^, which are essential for understanding metal homeostasis in Acanthocephala. Acanthocephalans, lacking a digestive system, absorb nutrients and accumulate metals, especially toxic ones^4,7,17–20^, likely due to competition for essential elements and enhanced uptake via bile salts, which form bioavailable metal complexes^17,21–24^. Reliant on host-derived steroids and fatty acids, they efficiently absorb bile salts, inadvertently taking up toxic metals^6,25,26^. Mechanisms of metal homeostasis remain unclear, with molecular data available only for a single acanthocephalan species, *Pomphorhynchus laevis*.

The identification of MBPs typically involves annotating assembled transcripts using databases such as PFAM, Swiss-Prot and NR (Non-Redundant) which combine sequence alignment, domain identification and functional annotations^27^. Predictive tools like MeBiPred, which assess metal-binding potential based on sequence information of proteins that interact with ubiquitous metal ligands such as zinc (Zn), copper (Cu), cobalt (Co) and others essential for life yet toxic in excess^28–31^. The identified MBPs could then be compared in different but evolutionarily related taxa, which would provide valuable insights into their functions, evolutionary conservation and adaptations.

This study aimed to assemble and analyze the *de novo* transcriptome of *D. truttae* with a focus on identifying orthologous genes and characterizing MBPs in detail. By generating the first publicly available transcriptome for the family Leptorhynchoididae Witenberg, 1932, this research provides a foundation for addressing questions related to the phylogeny, taxonomy, diversity and evolution of Acanthocephala. Additionally, the ecological roles of MBPs in metal accumulation and host–parasite interactions are explored, offering insights into the broader significance of *D. truttae* as both a bioindicator and a model species.

## Results

### *De Novo* assembly of the *D. truttae* transcriptome

A total of 102.66 Gb of clean reads (98.51% of raw reads) were generated from the eight samples of *D. truttae* (Table S1), with an average of 42.7 million clean reads and 34.2 million total mapped reads. The Trinity assembler generated 129,887 transcripts with an average length of 915 bp and an N50 of 1193 bp and a total of 61,570 unigenes with an average length of 817 bp and an N50 of 1027 bp. The total length of the transcriptome was 118,871,775 bp when considering the transcripts, whereas it was 50,302,772 bp when accounting for the unigenes. Most transcripts and unigenes were 300 bp to 500 bp in length (38.6% of transcripts and 47.1% of unigenes), and 8.9% of transcripts and 7.0% of unigenes were larger than 2,000 bp (Fig. S1). The longest sequence was 15,212 bp, and the shortest sequence was 301 bp for both the transcript and unigene databases. BUSCO assessment of the completeness of the *D. truttae* transcriptome assemblies revealed a high quality of the assembled transcripts (Fig. S2). The percentage of fragmented reads was low (3% for Trinity.fasta and 4.8% for unigene.fasta sequences), while the percentage of missing reads was higher (25.2% for Trinity.fasta and 26.8% for unigene.fasta sequences) (Fig. S2).

### Functional annotation

The statistics of the genes successfully annotated by each database can be found in Table 1, which shows that 40.6% of the unigenes were annotated in at least one database.

**Table 1:** Proportion of genes successfully annotated in the *D. truttae de novo* transcriptome across different databases with percentages calculated relative to the total unigene count.

	Number of unigenes	Percentage (%)
Total Unigenes	61,570	100.00
Annotated in at least one database	25,002	40.60
Annotated in PFAM (Protein family)	18,022	29.27
Annotated in GO	18,017	29.26
Annotated in NR	14,662	23.81
Annotated in SwissProt	8976	14.57
Annotated in KO (KEGG orthology)	7616	12.36
Annotated in KOG	6729	10.92
Annotated in NT (NCBI nucleotide sequences)	2806	4.55
Annotated in all databases	996	1.61

**Fig. 1:**
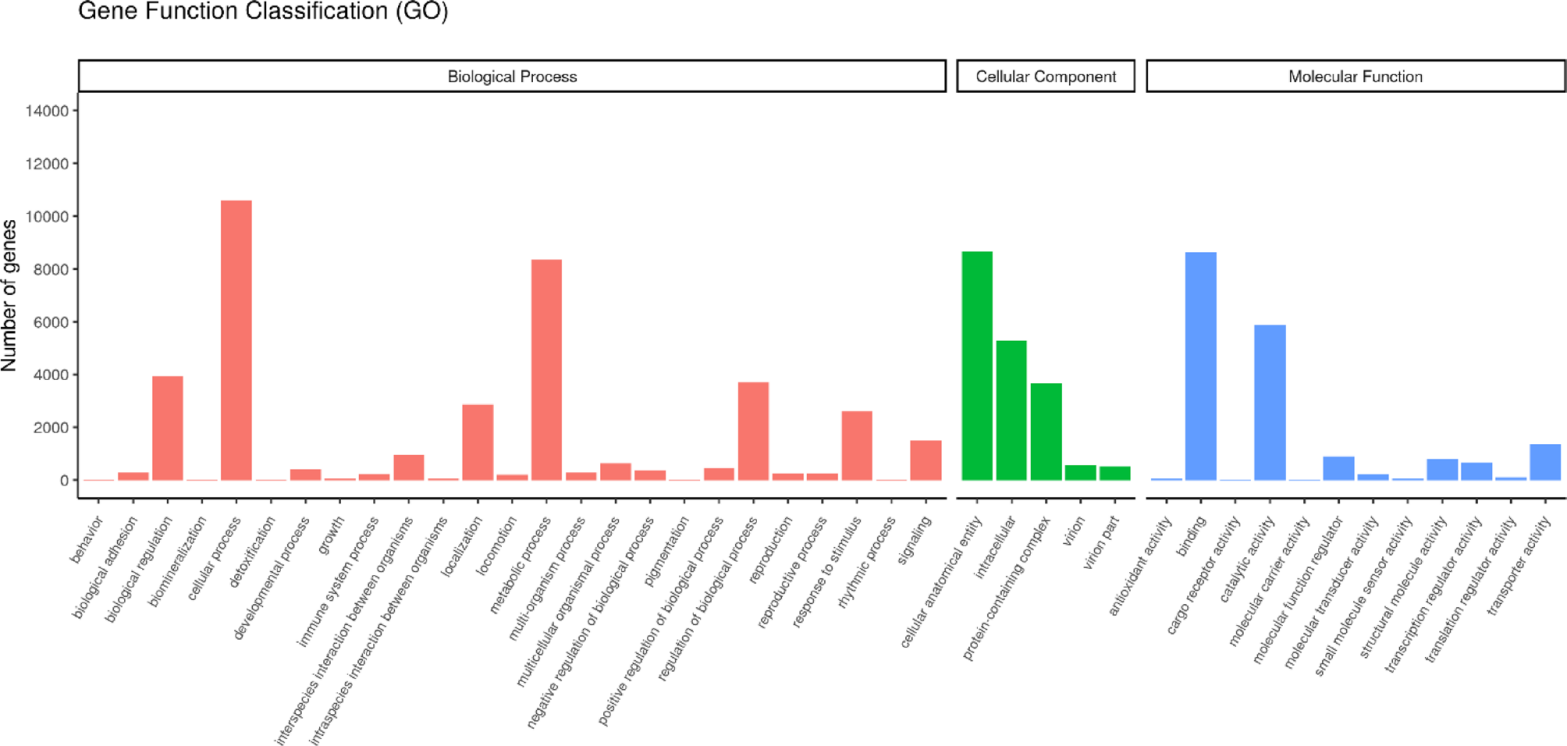
Classification of *D. truttae* unigenes annotated in the Gene Ontology (GO) database into three functional GO categories: biological process (BP), cellular component (CC) and molecular function (MF).

After GO analysis, a total of 18,017 unigenes were assigned to the three main GO categories: 50.4% to biological processes (BP), with 25 subcategories; 24.8% to cellular component (CC), with five subcategories; and 24.8% to molecular function (MF), with 12 subcategories (Fig. 1). The highest number of unigenes in BP was associated with cellular processes (27.9%), metabolic processes (22.0%) and biological regulation (10.4%). Further analysis of the biological processes at GO levels 3 and 4 showed that most of the genes were associated with metabolism and its regulation, organization and biogenesis of cellular components, biosynthesis, and regulation of response to stimuli, localization and transport. In the CC category, unigenes were abundant as cellular anatomical entities (46.3%), intracellular entities (28.3%) and protein-containing complexes (19.6%). Most of these genes were associated with organelles and membranes. Most of the unigenes in the MF category were associated with binding (46.3%), catalytic activity (31.5%) and transporter activity (7.2%) and were linked to the binding of nucleic acids, nucleoside phosphates, ions and signaling receptors, as well as to the activity of transferases, peptidases, hydrolases, and transmembrane transporters.

**Fig. 2:**
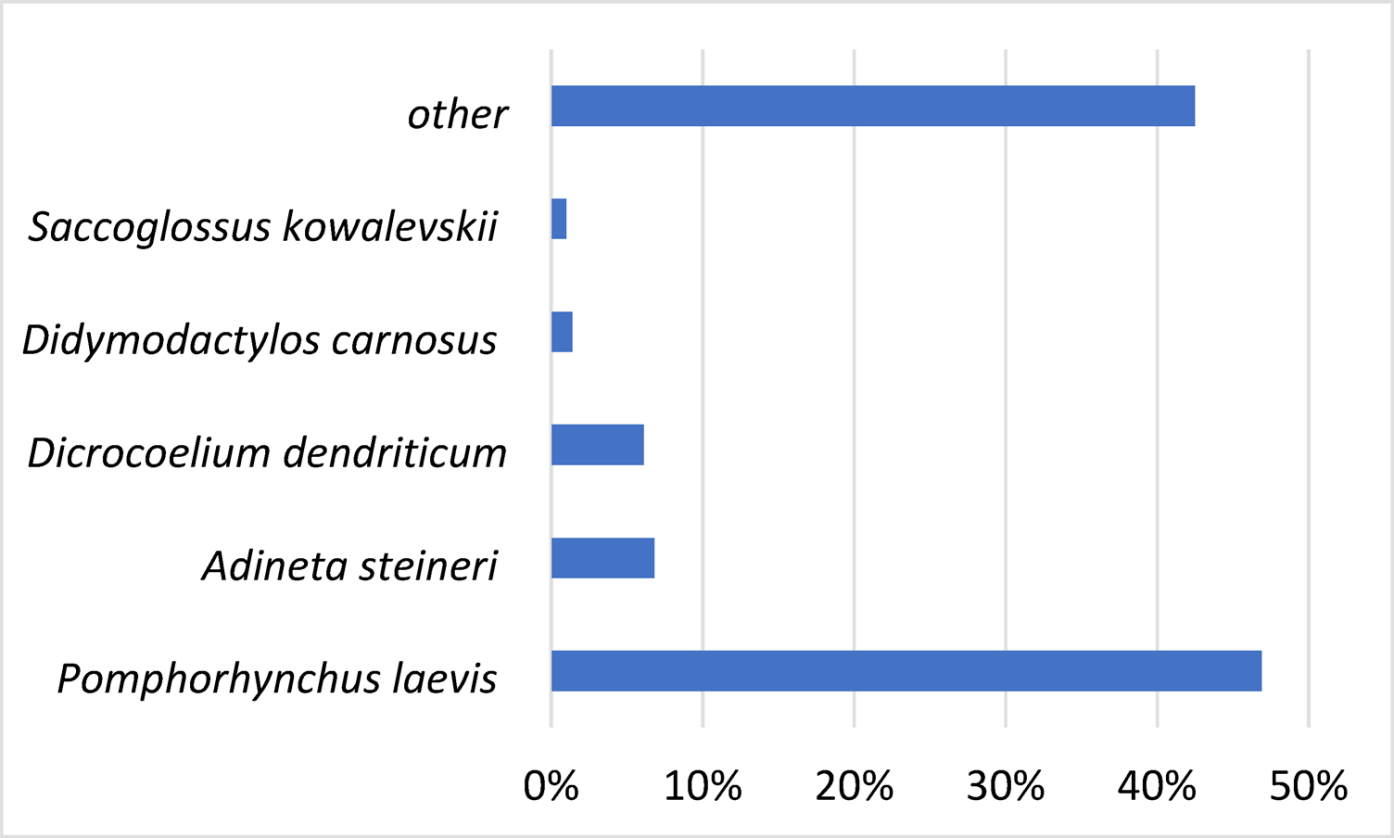
Species distribution of *D. truttae* unigenes annotated in the NR database (NCBI non-redundant protein sequences). This graph shows the percentage of unigenes that match sequences from different species showing the top five most represented species.

A total of 14,662 unigenes were annotated by matching them to the NR database and used to further determine the GO terms and KEGG pathways. According to the species distribution of the sequences annotated in the NR database, 46.9% of the matched unigenes showed similarities with sequences of *Pomphorhynchus laevis* (Acantocephala), followed by *Dicrocoelium dendriticum* (Platyhelminthes, Trematoda) (6.1%), *Didymodactylos carnosus* (Rotifera) (1.4%), *Adineta steineri* (Rotifera) (6.8%), *Saccoglossus kowalevskii* (Hemichordata) (1.0%), *Rotaria magnacalcarata* (Rotifera) (1.0%), and others (42.5%) (Fig. 2).

Only a small proportion of unigenes matched sequences from the fish host *Salmo trutta* (0.91%), with an additional 0.91% matched sequences from *Salmo salar* and 0.53% from *Oncorhynchus mykiss*. This suggests that contamination with DNA from parasite hosts was not significant.

According to the KOG function classification, 6,729 of 14,662 unigenes (45.9%) were annotated and classified into 25 functional categories based on their predicted functions. Among them, the largest group was “General function prediction only” (11.2% genes), followed by “Signal transduction mechanisms” (11.1%), “Posttranslational modification, protein turnover, chaperones” (10.4%), “Translation, ribosomal structure and biogenesis” (8.5%) and “Transcription” (6.8%) (Fig. S3). The least represented categories were “extracellular structures” (0.4%) and “cell motility” (0.2%).

**Fig. 3:**
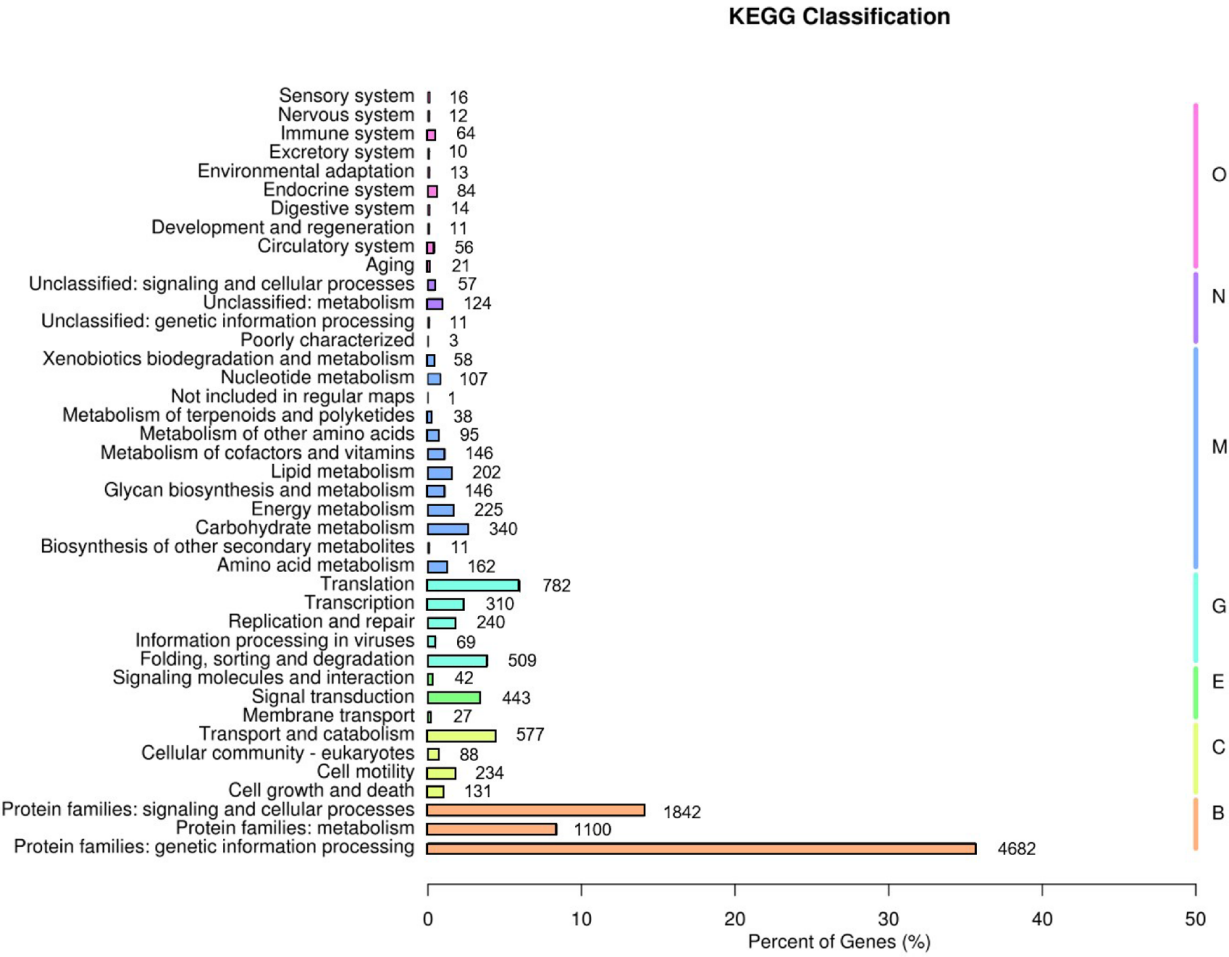
Functional classification of annotated genes based on the KEGG database^68–70^. KEGG functional categories are grouped into two major classifications: KEGG Pathways, which include categories such as Metabolism (M), Genetic Information Processing (G), Environmental Information Processing (E), Cellular Processes (C), Organismal Systems (O), and Not Included in Pathway or BRITE (N), and BRITE Hierarchy, which includes higher-level functional groupings such as “Protein families: metabolism”, “Protein families: genetic information processing” and “Protein families: signaling and cellular processes” (represented by code B).

A total of 7,616 out of 14,662 unigenes (51.9%) were assigned to 303 KEGG pathways belonging to seven KO pathway level 1 categories (markers B, C, E, G, M, N, and O in Fig. 3). Most genes (58.2%) belonged to “BRITE Hierarchies” (B), while only 1.2% belonged to “Not Included in Pathway or BRITE” (N) (Fig. S4).

Most of the unigenes in the largest subcategory of the “BRITE Hierarchies”, “Genetic information processing”, were included in the pathways “Membrane trafficking” (15.9%), “Chromosome and associated proteins” (11.9%), “Messenger RNA biogenesis” (8.0%) and “Spliceosome” (7.9%) (Fig. S5). The pathways that contained the largest number of unigenes in the category “Signaling and Cellular Processes” were “Exosome” (31.3%) and “Cytoskeleton proteins” (17.2%), while those in the “Metabolism” category were “Peptidases and inhibitors” (26.3%), “Protein phosphatases and associated proteins” (25.1%) and “Protein kinases” (25.0%). Within “Genetic Information Processing” category the subcategory “Translation” contained the most unigenes associated with the KEGG pathways “Ribosome” (39.4%), “Ribosome biogenesis in eukaryotes” (21.0%) and “mRNA surveillance pathway” (17.2%).

### Identification of orthologous sequences

OrthoFinder has been used to identify orthogroups, i.e. groups of genes that share a common ancestor and provide important insights into gene function, evolutionary history and species adaptations among *D. truttae*, *Pomphorhynchus laevis* (Acanthocephala), *Adineta steineri*, *Rotaria socialis*, *Brachionus calyciflorus* (Rotifera), *Ancylostoma ceylanicum*, *Trichinella nativa* (Nematoda), *Dicrocoelium dendriticum* (Platyhelminthes, Trematoda) and *Saccoglossus kowalevskii* (Hemichordata). The analysis assigned 169,531 genes from these nine species (66.1% of the total) to 49,452 orthogroups. Half of the assigned genes were present in orthogroups with four or more genes (G50 = 4) and were contained in the largest 12,529 orthogroups (G50 = 12,529), emphasizing the comprehensive nature of the orthogroup assignments and their evolutionary significance.

While over 80% of genes in *A. steineri* (83.2%), and *A. ceylanicum* (81.7%) were assigned to orthogroups, more than half of the genes in *P. laevis* (50.8%), *B. calyciflorus* (57.3%), and *S. kowalevskii* (68.7%) remained unassigned (Table 2, Fig. S6), highlighting differences in gene assignment rates and the varying degrees of genomic characterization among species.

**Table 2:** Species-specific statistics of orthofinder orthogroup analysis among coding sequences of *D. trutta* and eight phylogenetically or lifestyle-related species with reference genomes available in the NCBI database: *Pomphorhynchus laevis*, *Adineta steineri*, *Rotaria socialis*, *Brachionus calyciflorus*, *Ancylostoma ceylanicum*, *Trichinella nativa*, *Dicrocoelium dendriticum* and *Saccoglossus kowalevskii.*

	Acanthocephala		Rotifera			Nematoda		Platyhelminthes	Hemichordata
	*D. truttae*	*P*. laevis	*A. steineri*	*R*. socialis	*B. calyciflorus*	*A. ceylanicum*	*T. nativa*	*D. dendriticum*	*S. kowalevskii*
Number of genes	**13,457**	13,055	50,525	33,717	24,404	65,583	16,586	16,907	22,132
Number of genes in orthogroups	**10,345**	6423	42,052	20,137	10,413	53,574	10,603	9055	6929
Number of unassigned genes	**3112**	6632	8473	13,580	13,991	12,009	5983	7852	15,203
% of genes in orthogroups	**76.9**	49.2	83.2	59.7	42.7	81.7	63.9	53.6	31.3
% of unassigned genes	**23.1**	50.8	16.8	40.3	57.3	18.3	36.1	46.4	68.7
Number of orthogroups containing species*	**3512**	1875	17,845	13,944	3812	13,538	3088	2610	2456
% of orthogroups containing species	**7.1**	3.8	36.1	28.2	7.7	27.4	6.2	5.3	5.0
Number of species-specific orthogroups	**3432**	1737	5734	1828	3224	13,438	2938	2550	2209
Number of genes in species-specific orthogroups	**10,144**	6214	17,858	5274	9623	53,337	10,324	8960	6565
% of genes in species-specific orthogroups	**75.4**	47.6	35.3	15.6	39.4	81.3	62.2	53.0	29.7

*Number of orthogroups containing species – total number of orthogroups in which the species listed at the top of the column is present.

Only two orthogroups contained genes from all nine species, and neither of these was a single-copy orthogroup. The majority of orthogroups were species-specific, with 37,090 orthogroups (75.0%) containing 128,299 genes (50.0% of the total). The number of species-specific genes ranged from 5,274 (15.6%) in *R. socialis* to 53,337 (81.3%) in *A. ceylanicum* (Table 2, Fig. S6), illustrating the considerable variability in gene distribution and species-specific evolution across the dataset.

The highest number of shared orthogroups was observed among the rotifer species and between them and *S. kowalevskii* (Hemichordata) (Table 3). Notably, 10,144 (75.4%) *D. truttae* genes were found in species-specific orthogroups, with only 1.5% shared across different species. The species sharing the highest proportion of orthogroups with *D. truttae* were *P. laevis* (20.8% of shared orthogroups), *A. steineri* (16.1%), *R. socialis* (16.1%) and *A. ceylanicum* (14.8%) (Fig. S7), highlighting patterns of gene conservation and divergence across species.

The shared orthogroups provide valuable insights into evolutionary relationships and functional similarities, helping to distinguish conserved genes that are shared across species from species-specific genes that reveal evolutionary pressures and functional innovations. For instance, the extensive sharing of orthogroups among rotifers and *S. kowalevskii* suggests conserved functional roles and evolutionary constraints linked to their shared aquatic habitats and developmental biology. Among the species analyzed, *A. steineri* and *R. socialis* shared the highest number of orthogroups (12,768), reflecting their close phylogenetic relationship within Bdelloidea rotifers and high genomic similarity. Conversely, the lower level of shared orthogroups between *P. laevis* and *D. truttae*, despite their phylogenetic proximity as parasitic organisms, suggests divergent evolutionary pressures potentially related to host specialization and life cycle adaptations.

Interestingly, *P. laevis* shared more orthogroups with rotifers than with *D. truttae*, which contrasts with expected phylogenetic relationships and ecological similarities. This pattern indicates that evolutionary convergence in certain genes related to environmental adaptation may have occurred between *P. laevis* and the rotifers. Furthermore, the identification of 12 *P. laevis* genes with multiple orthologs in *D. truttae* implies post-speciation gene duplication events, contributing to potential functional diversification.

Orthogroup analysis thus highlights not only evolutionary ancestry but also gene family expansions, contractions, and functional adaptations specific to ecological niches, demonstrating its importance for comparative genomics by helping researchers understand how gene duplication, loss, and retention shape genomes over time. The distribution of orthogroups across species emphasizes both conserved genetic pathways and species-specific innovations that reflect adaptive strategies within their respective clades.

**Table 3:** Number of shared orthogroups among the acanthocephalans *Dentitruncus truttae* and *Pomphorhynchus laevis* and other species related by phylogeny or lifestyle.

	Acanthocephala		Rotifera			Nematoda		Hemichordata	Platyhelminthes
	*D. truttae*	*P*. laevis	*A. steineri*	*R*. socialis	*B. calyciflorus*	*A. ceylanicum*	*T. nativa*	*S. kowalevskii*	*D. dendriticum*
***D. truttae***		**31**	**24**	**24**	**13**	**22**	**15**	**11**	**9**
*P. laevis*			78	82	55	8	14	30	10
*A. steineri*				12,768	490	38	76	157	27
*R. socialis*					488	37	79	152	27
*B. calyciflorus*						19	59	94	14
*A. ceylanicum*							22	26	14
*T. nativa*								29	12
*S. kowalevskii*									20
*D. dendriticum*									

### Metal-binding proteins

**Fig. 4:**
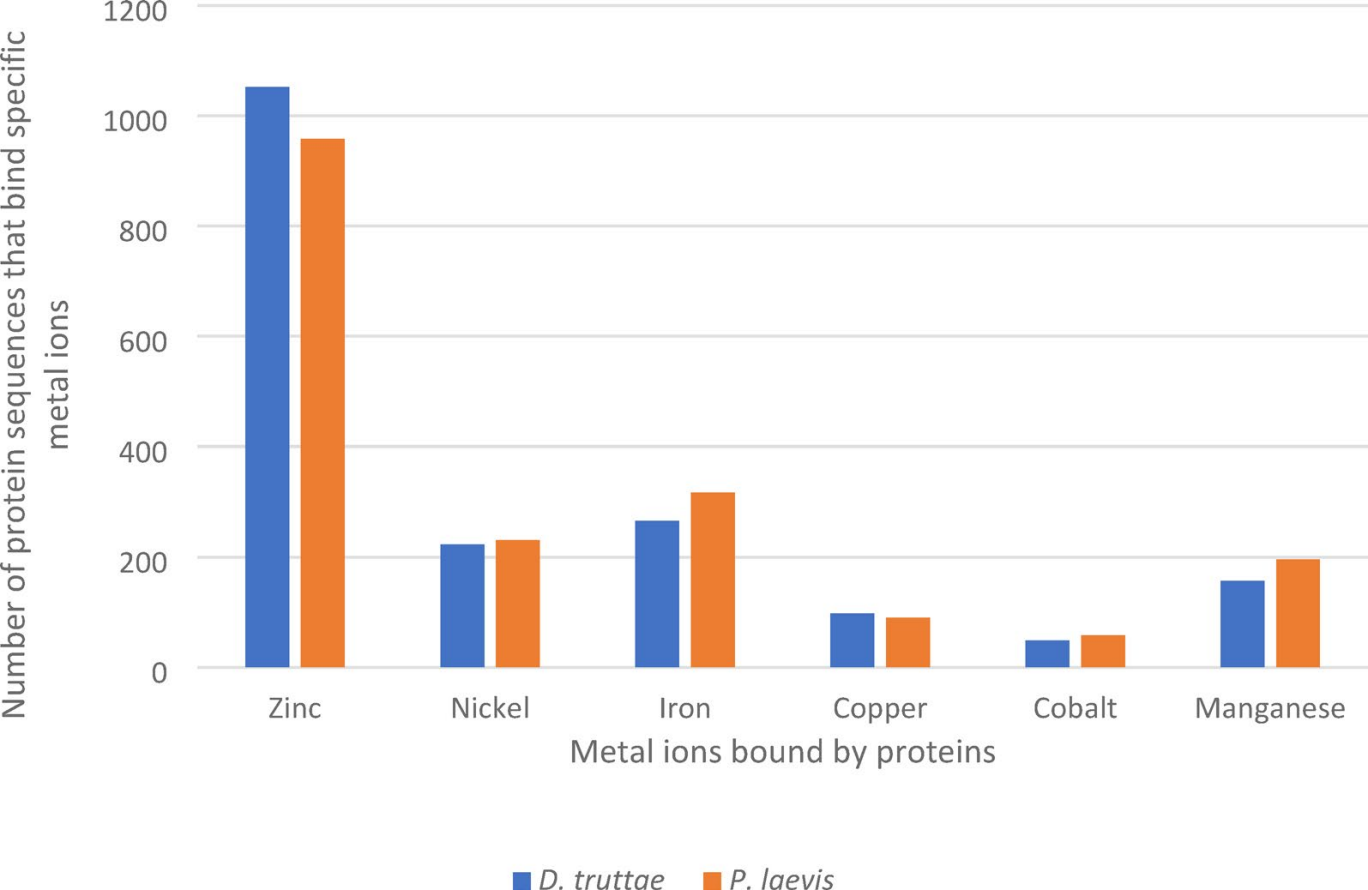
Comparison of the number of metal-binding proteins that bind different metal ions (zinc, nickel, iron, copper, cobalt, manganese) in two Acanthocephala species, *D. truttae* and *P. laevis*, according to the MeBiPred analysis.

After the entire proteome of *D. truttae* (13,457 protein sequences) was submitted to the MeBiPred server for prediction of metal-binding properties, 1,944 protein sequences (14.5%) were characterized as metal-binding (Supplementary File 2, Supplementary File 2a), with a remarkable distribution across different metal ions. Zinc-binding proteins were the most abundant (53.8%, 1,052 proteins), followed by iron- (13.6%, 266 proteins) and nickel-binding proteins (11.4%, 233 proteins) (Fig. 4). A relatively low number of copper- (5.0%, 98 proteins) and cobalt-binding proteins (2.5%, 49 proteins) were predicted, while manganese-binding proteins bind to 8.0% (157 proteins) of MBPs in *D. truttae* (Fig. 4). Proteomic analysis of *P. laevis* (GCA_012934845.2) showed a similar metal-binding pattern, with 15.5% (2,029 protein sequences) of the proteome exhibiting metal-binding properties (Supplementary File 2, Fig. 4 Supplementary File 2b). Zinc-binding proteins were also the most abundant MBPs (47.2%, 958 proteins), followed by iron- (15.6%, 317 proteins) and nickel- (11.4%, 231 proteins) binding proteins, with a similar low prediction of manganese- (9.7%, 196 proteins), copper- (4.4%, 90 proteins) and cobalt- (2.9%, 58 proteins) ion-binding proteins. However, 796 of the total MBPs in *D. truttae* were predicted to bind two or more ions, compared to 832 in *P. laevis* (Supplementary File 2).

**Fig. 5:**
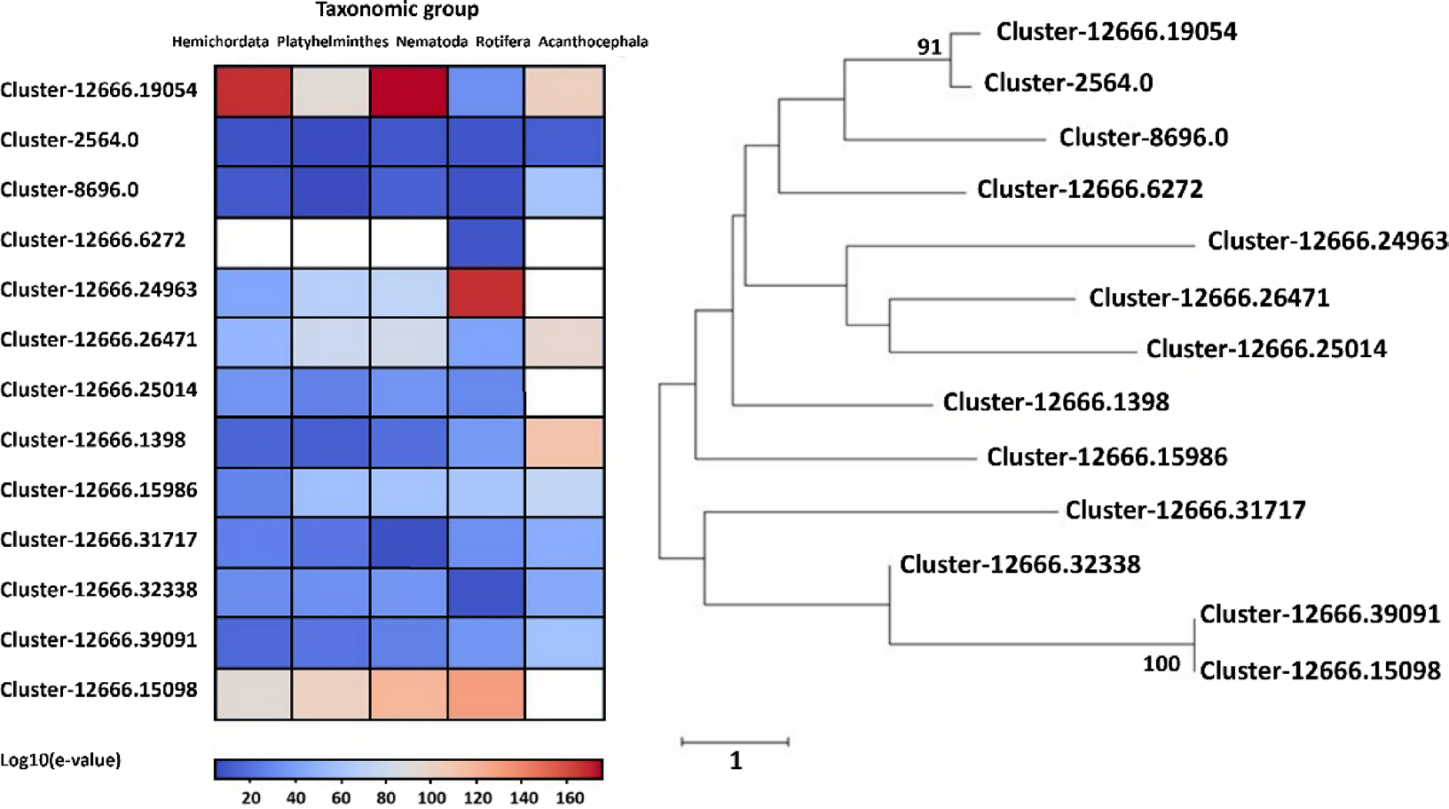
Phylogenetic analysis of *D. truttae* zinc metalloproteases using 1000 replicates and Whelan Goldman + Freq Model. Only bootstrap values ≥ 70% are shown. The tree is drawn to scale, with branch lengths representing the number of substitutions per site, as indicated by the scale bar. The heatmap represents log10-transformed e-values from alignments against sequences from Acanthocephala, Rotifera, Nematoda, Platyhelminthes, and Hemichordata, with red indicating significant alignments and blank boxes representing no similar sequences.

Of the 1,944 protein sequences predicted as MBPs by the MeBiPred software, 1,383 were described by the PFAM database (e-value threshold is 0.01) as having specific protein domains. Of these, 265 were associated with binding of zinc ions (Supplementary File 3), 23 with nickel ions (Supplementary File 4), 22 with copper ions (Supplementary File 5), 11 with iron ions (Supplementary File 6) and no protein sequence was associated with a specific domain for cobalt binding. When we analyzed the zinc-binding proteins in *D. truttae*, the majority (246 protein sequences) was classified as zinc finger proteins and 13 as metalloproteases (Supplementary File 3, Fig. 5). Zinc fingers, a common motif, are particularly involved in gene regulation, which may explain their prevalence among the proteins in these organisms. However, as metalloproteases are a more homogeneous protein group, we further analyzed 13 protein sequences from *D. truttae* for their similarity and agreement with the NCBI GenBank data (Fig. 5). These proteins could support nutrient acquisition and stress resistance by breaking down host or environmental proteins, managing oxidative stress, and adapting to metal-rich environments, potentially enhancing parasite survival. The phylogenetic tree showed a division of the sequences into five not clearly delimited clusters consisting of matrixin, reprolysin and some proteins with functions unknown by PFAM (Supplementary File 3, Fig. 5). The heatmap results showed that the metalloproteases of Acanthocephala (mainly matrixins and reprolysins) are highly conserved with *P. laevis* (Fig. 5). In cases where the NCBI database did not contain data for *P. laevis* homologues, the metalloprotease sequences of *D. truttae* showed more similarities with evolutionarily close Rotifera and parasitic Nematoda. Only one sequence (Cluster-12666.19054) was an exception, showing the highest degree of similarity to Nematoda and Hemichordata. These proteins may play a crucial role in nutrient acquisition and stress resistance by degrading host tissue and remodeling of extracellular matrix components.

**Fig. 6:**
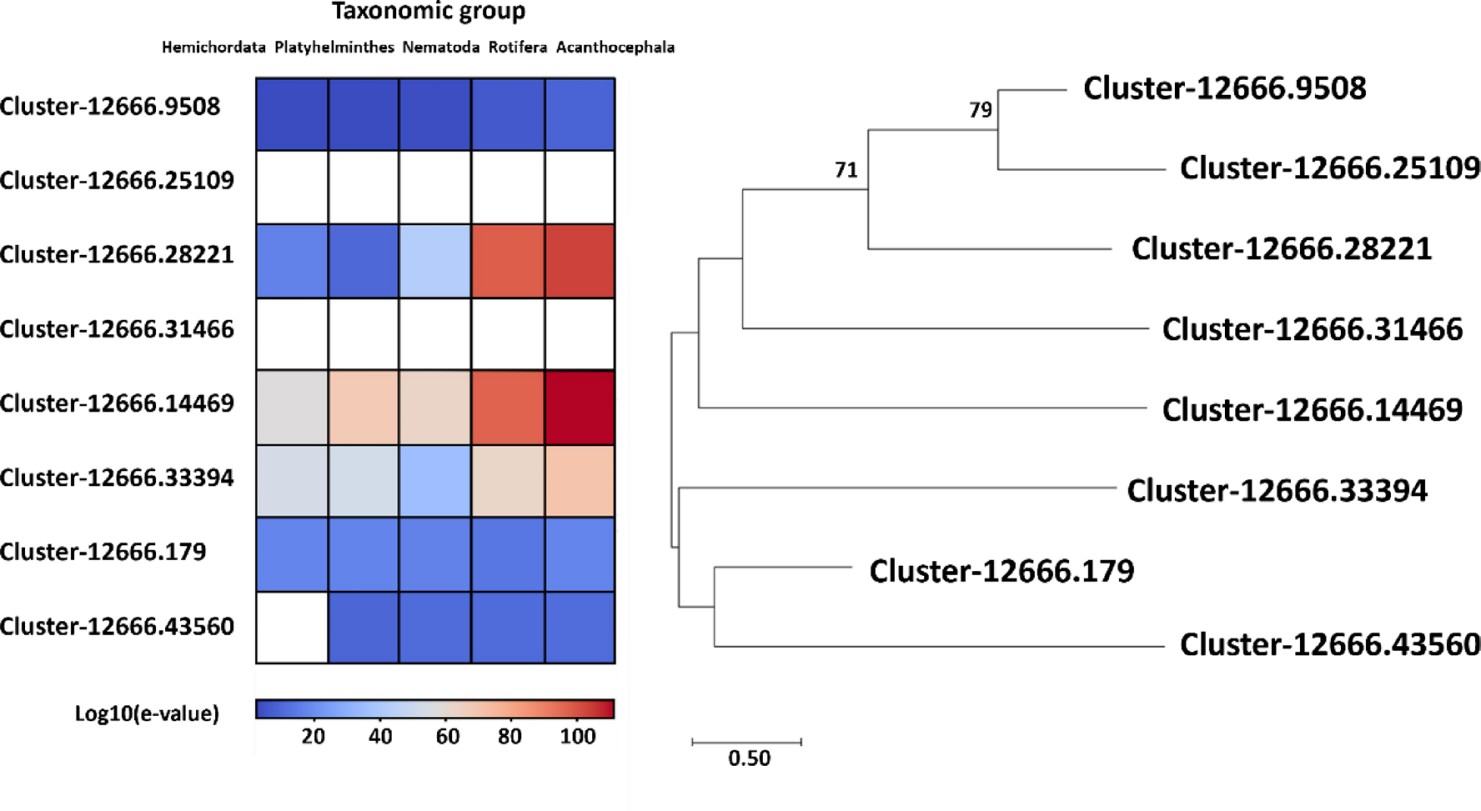
Phylogenetic analysis of *D. truttae* iron-sulfur proteins using 1000 replicates and Whelan Goldman + Freq Model. Only bootstrap values ≥ 70% are shown. The tree is drawn to scale, with branch lengths representing the number of substitutions per site, as indicated by the scale bar. The heatmap represents log10-transformed e-values from alignments against sequences from Acanthocephala, Rotifera, Nematoda, Platyhelminthes, and Hemichordata, with red indicating significant alignments and blank boxes representing no similar sequences.

 Nineteen sequences have been described that bind iron, eight of which have been identified as the iron-sulfur group (Supplementary File 6, Fig. 6). These iron-sulfur binding and zinc-finger proteins may contribute to parasitic adaptation by facilitating survival in metal-rich host environments, possibly through improved redox homeostasis and protection against oxidative stress. When we analyzed these sequences in a phylogenetic tree, two main clusters emerged (Fig. 6). One cluster consisted of two proteins with predicted NADH-ubiquinone oxidoreductase-G iron-sulfur binding region (Cluster-12666.43560 and Cluster-12666.33394) and one zinc-finger (Cluster-12666.179). Only one sequence (Cluster-12666.33394) showed a higher degree of similarity to other Acanthocephala species (P.laevis) and Rotifera, shown in red squares in the heatmap. Other cluster contained more variable sequences, two of which (Cluster-12666.14469 and Cluster-12666.28221) showed the highest similarity to P.laevis and Rotifera.

**Fig. 7:**
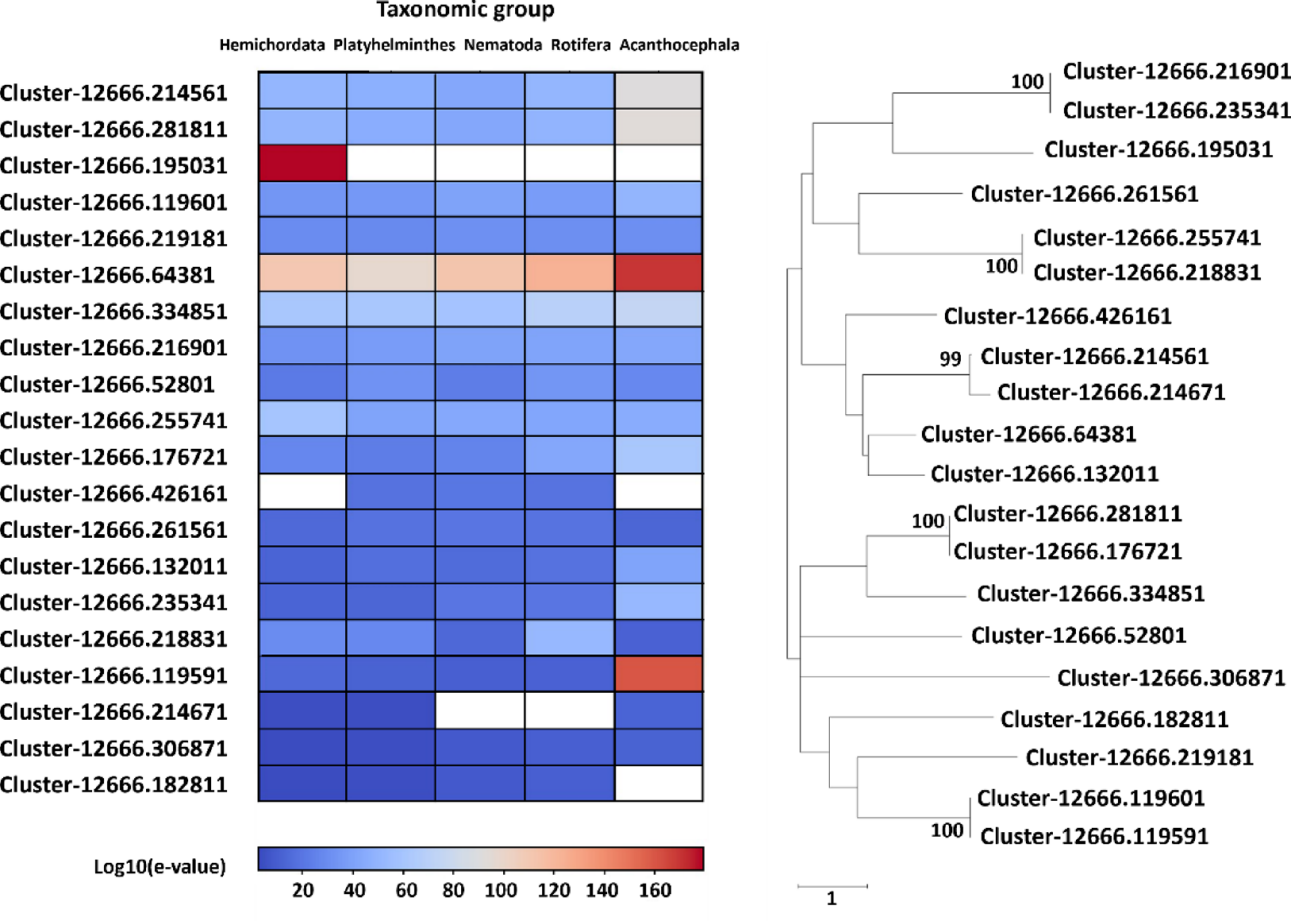
Phylogenetic analysis of *D. truttae* nickel urease/hydrogenase proteins using 1000 replicates and Whelan Goldman + Freq Model. Only bootstrap values ≥ 70% are shown. The tree is drawn to scale, with branch lengths representing the number of substitutions per site, as indicated by the scale bar. The heatmap represents log10-transformed e-values from alignments against sequences from Acanthocephala, Rotifera, Nematoda, Platyhelminthes, and Hemichordata, with red indicating significant alignments and blank boxes representing no similar sequences.

When we analyzed the nickel-binding proteins in *D. truttae*, 23 were PFAM described as nickel ureases/hydrogenases (Supplementary File 4). The phylogenetic tree, which consisted of similar protein sequences from invertebrates, showed three major clusters that are not clearly variable in the PFAM definition (Fig. 7). The heatmap showed the highest degree of similarity with other Acanthocephala (*P. laevis*) and Rotifera, and one sequence (Cluster-12666.195031) had no similar homologues other than the taxonomic group Hemichordata, with a high degree of similarity (Fig. 7). These nickel-binding proteins likely support parasite adaptation by contributing to nitrogen metabolism and mitigating the toxic effects of metal accumulation by enhancing survival in metal-rich host environments through processes such as urease-driven ammonia detoxification and redox balance maintenance.

Of the 19 sequences identified as potential invertebrate metallothioneins by HMMER using the PFAM database, five were characterized as MBPs by MeBiPred (Supplementary File 7). Of these five proteins, one was identified by the NR database as most similar to *Pomphorhynchus laevis* (Acanthocephala), two had identical amino acid sequences and were most similar to *Trichinella patagoniensis* (Nematoda), one was most similar to *Branchiostoma belcheri* (Cephalochordata) and one sequence showed no significant similarity in blasting to a sequence in the NCBI database.

Interestingly, the protein sequence that was most similar to *T. patagoniensis* (55.6%) also had the highest similarity to plant metallothioneins (87.7% and 86.1% for *Carpus fangiana* and *Corylus avellane*, respectively). No other organisms, except plants and *T. patagoniensis*, showed such a high degree of similarity. Metallothioneins are known for their chelating properties for metal ions, which could help parasites such as *P. laevis* and *T. patagoniensis* to cope with metal toxicity while contributing to redox regulation and resistance to oxidative stress during parasitism.

**Fig. 8:**
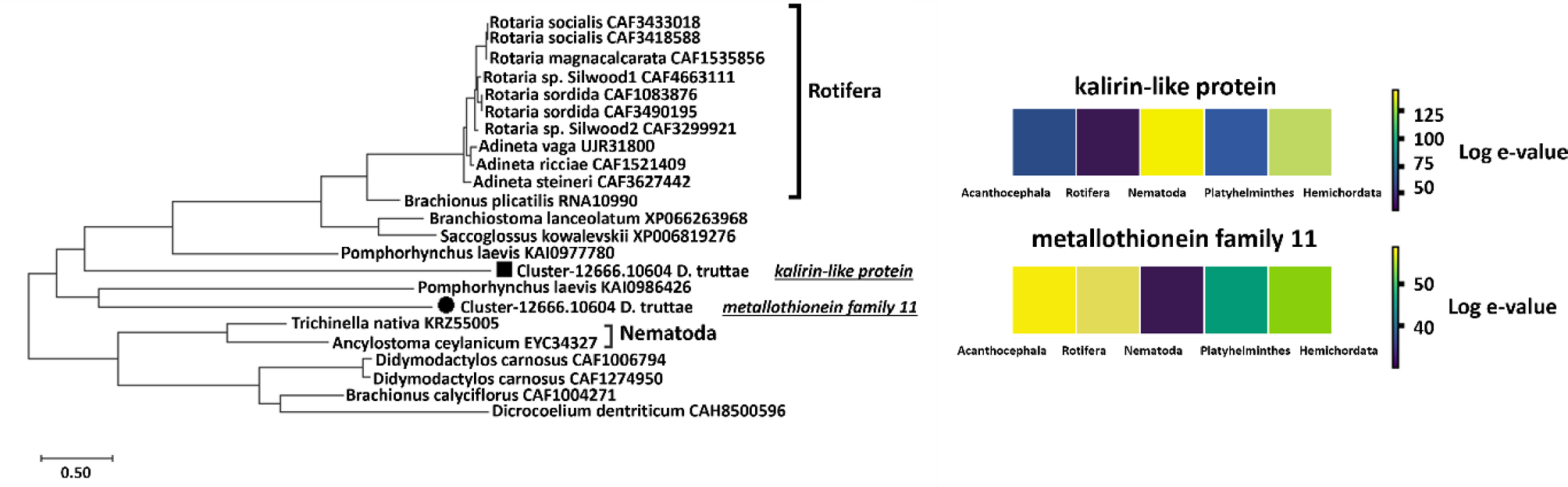
Phylogenetic analysis of two *D. truttae* metallothioneins using 1000 replicates and JTT matrix-based Model. The threshold for displaying bootstrap support values was 70%. The tree is drawn to scale, with branch lengths representing the number of substitutions per site, as indicated by the scale bar. The black circle and square in the tree indicate two *D. truttae* metallothionein protein sequences which were identified as kalirin-like protein and a member of metallothionein family 11. The heatmap represents log10-transformed e-values from alignments against sequences from Acanthocephala, Rotifera, Nematoda, Platyhelminthes, and Hemichordata, with purple indicating significant alignments and yellow boxes representing less matching sequences.

To further explore evolutionary relationships, we constructed a phylogenetic tree and heatmap for the remaining two metallothionein sequences. The results confirmed their highest similarity with sequences from the closely related acanthocephalan *P. laevis* and various species of Rotifera (Fig. 8). These observations support the hypothesis that metallothioneins are critical for survival in dynamic, metal-rich environments and contribute to the evolutionary success of parasitic species inhabiting a range of ecological niches.

**Fig. 9:**
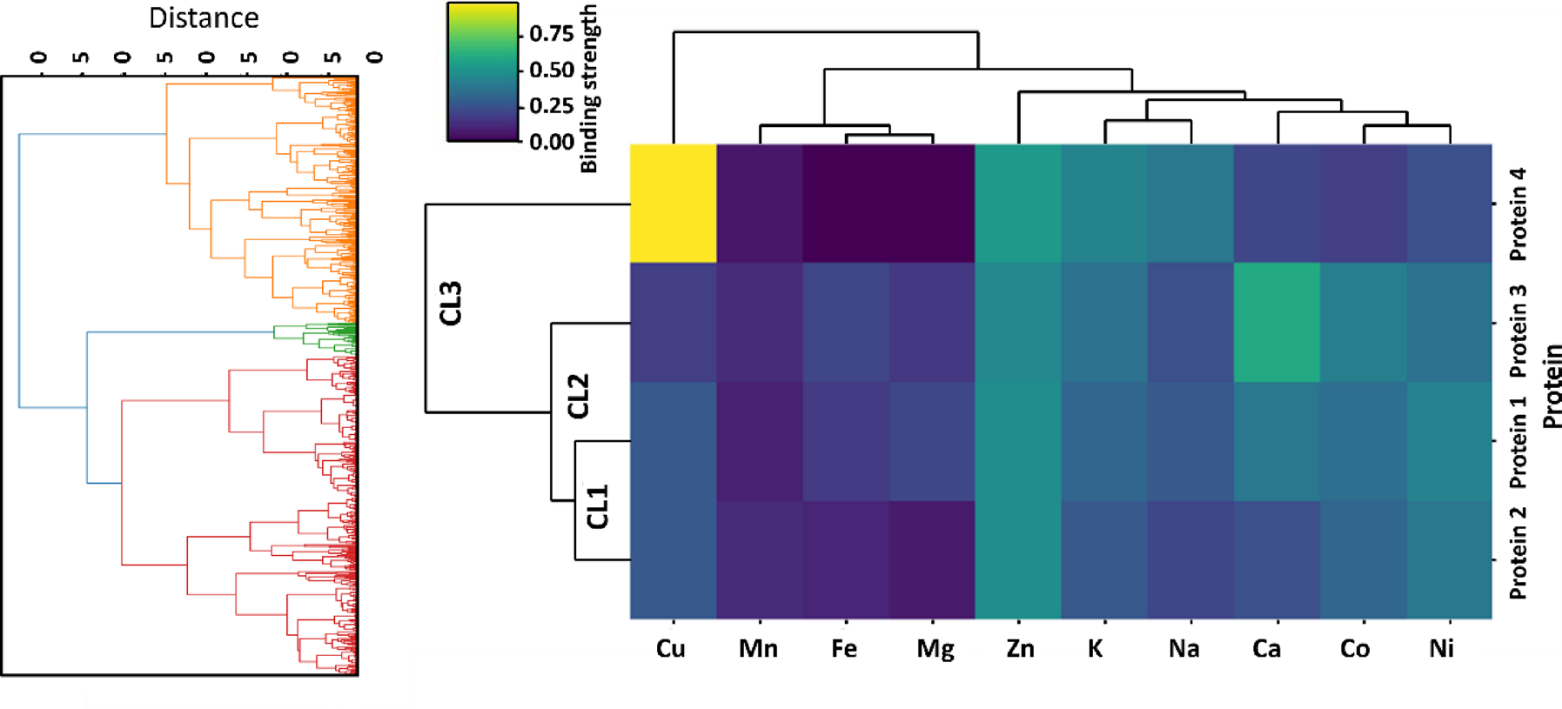
Hierarchical clustering heatmap of *D. truttae* metal-binding proteins lacking PFAM annotation, grouped into three clusters (CL1, CL2, and CL3) based on predicted metal-binding affinities. Binding strength values are color-coded, ranging from low (dark purple) to high (yellow), as indicated by the scale. The dendrograms represent hierarchical relationships among both proteins (right) and metal ions (top), based on binding preferences for Cu, Mn, Fe, Mg, Zn, K, Na, Ca, Co, and Ni (Supplementary File 8).

To group the remaining 561 protein sequences for which we could not find characteristic metal-binding domains in the PFAM database, we categorized them according to the predictions of the MeBiPred software for specific metal binding (Fig. 9). The hierarchical cluster map visualized patterns in their metal binding preferences, based on which these 561 proteins were divided into three main clusters, the largest of which is subdivided into two subclusters (Fig. 9, Supplementary File 8). These two subclusters (protein 1 and 2 in Fig. 9) showed similar binding preferences, characterized by higher binding of Zn, Ni, Co and Cu ions, while lower binding preferences were observed for Mn and Mg ions. The members of the second cluster were characterized by the highest preference for the binding of Ca and the lowest for the binding of Mn. The third cluster consisted of proteins with a high preference for binding Cu and a low preference for binding Fe and Mg ions (Fig. 9). It is evident that all clusters had an almost equal preference for binding Zn ions, while Mg, Fe and Mn were bound with the lowest preference.

To refine our analysis of metal-binding proteins (MBPs), we focused on protein groups with specific metal-binding domains using HMMER (HMMER package, Seed Alignment, e-value threshold of 1e^− 5^). The analysis revealed that most MBPs in *D. truttae* belonged to the PFAM family “ABC transporter”, with seven sequences, followed by "solute carrier family 39 member 10", "Ctr copper transporter family", “Metallothionein”, and "ABC transporter transmembrane region" (two sequences each) and "Cation efflux family", "Inositol polyphosphate kinase", and "Glutathione S-transferase, N-terminal domain" (one sequence each).

Comparative analysis with organisms from Rotifera, Nematoda, Trematoda and Hemichordata revealed the greatest similarities between the acanthocephalans *D. truttae* and *P. laevis*, and between members of Nematoda (*Trichinella nativa* and *Trichinella patogoniensis*) and Trematoda (*Dicrocoelium dendriticum*). Members of the phylum Rotifera had a significantly higher number of genes (Table 2) and consequently a higher number of proteins belonging to the analyzed PFAM groups containing metal-binding domains (Table 4, Supplementary File 9). The PFAM group “ABC transporter” consistently contained the highest number of proteins. However, only a small proportion (11.0-24.0%) were predicted as MBPs by the MeBiPred software (Table 4, Supplementary File 9).

Interestingly, *D. truttae* possessed two MBPs from the "Ctr copper transporter family", a feature it shared with *Brachionus plicatilis* and *Saccoglossus kowalevskii*, whereas organisms with larger genomes generally possessed only one or no protein from this family (Table 4). Remarkably, *D. truttae* also contained an MBP from the “metallothionein” family, which was absent in the other organisms analyzed (Supplementary File 9).

In the "Cation efflux family", *P. laevis* contained proteins that were characterized as metal tolerance proteins in the Swiss-Prot database but were classified as non-metal-binding proteins by MeBiPred (clusters 12666.22969, 12666.23553, 12666.31057 and 12666.31058). In addition, no MBPs from *D. truttae* were identified within the family "Heavy-metal-associated domain", a result consistent with most of the parasitic organisms analyzed (Table 4).

One MBP belonging to the family "ABC transporter transmembrane region" (ATP-binding cassette) was identified in *D. truttae* and showed high sequence similarity to the hypothetical protein GJ496_010402 from *P. laevis*. However, this protein was predicted by MeBiPred to be non-metal-binding (Table 4, Supplementary File 2).

**Table 4:** Number of MBPs confirmed by the MeBiPred in selected metal-binding PFAM groups in Acanthocephala and related species.

PFAM groups		Acanthocephala		Rotifera				Nematoda			Platyhelminthes	Hemichordata
Name	PFAM no.	*Dentitruncus truttae*	*Pomphorhynchus laevis*	*Adineta steineri*	*Rotaria socialis*	*Brachionus calyciflorus*	*Brachionus plicatilis*	*Ancylostoma ceylanicum*	*Trichinella nativa*	*Trichinella patagoniensis*	*Dicrocoelium dendriticum*	*Saccoglossus kowalevskii*
		Number of metal-binding proteins within PFAM group/number of PFAM group members										
ABC transporter	PF00005	**7/46**	3/36	72/299	21/179	17/113	11/93	30/238	11/46	8/58	8/51	13/118
Cation efflux family	PF01545	**1/13**	0/7	11/48	0/32	1/15	1/15	12/23	1/12	0/7	2/6	2/10
ZIP Zinc transporter	PF02535	**2/12**	2/15	6/32	4/26	0/15	1/17	10/52	10/19	7/15	1/12	1/21
Ctr Copper transporter family	PF04145	**2/3**	0/1	0/12	0/8	1/5	2/5	0/15	0/1	0/1	0/2	2/7
P5-type ATPase cation transporter	PF12409	**0/1**	0	0/1	0/1	0/5	0/2	0/6	0/1	0/3	1/1	0/2
Ferritin-like domain	PF00210	**0/13**	1/3	0/5	0/7	0/15	0/11	0/3	0/1	0/1	0/3	0/4
Metallothionein	PF01439	**1/1**	0	0	0	0	0	0	0	0/1	0	0
Copper permeases	PF00403	**0/2**	0/1	0/6	1/7	0/3	0/2	5/8	0/3	0/6	0/4	1/7
Sco1 chaperons	PF03770	**1/4**	0/4	0/8	1/5	0/2	0/2	1/8	1/3	3/6	2/6	0/4
ATP-binding cassette	PF00664	**2/17**	0/9	49/153	2/55	2/41	3/35	6/116	1/10	1/20	0/18	3/53
Glutathione S-transferase	PF02798	**0/16**	0/17	2/83	3/33	1/27	0/27	0/35	2/6	3	2/22	8/54

## Discussion

As the entire transcriptome has only been published for a single species from the order Acanthocephala, the transcriptome sequence of *D. truttae* represents a valuable resource to advance research and improve our understanding of this group of parasites. We have particularly emphasized their physiologically fascinating and ecologically important ability to accumulate high concentrations of metals, the molecular mechanism of which is not yet understood. The *D. truttae* transcriptome, based on unigenes, spans 50.3 million bp, which exceeds the lengths reported for the transcriptomes of the acanthocephalan (*P. laevis*, 33.8 million bp) and two rotifers (*Brachionus manjavacas*, 40.1 million bp; and *Rotaria magnacalcarata*, 40.0 million bp) - based on filtered contigs^32^. Differences in the length distribution of transcripts and unigenes are to be expected due to the limitations of sequencing technology and biological variability^33^. The low percentage of fragmented reads in our assembly indicates that reconstruction successfully captured most of the expected single-copy orthologs. However, the relatively high percentage of missing reads may reflect transcript variability or species-specific factors in this *de novo* transcriptome analysis.

As expected, a higher proportion of *D. truttae* unigenes (40.6%) were annotated in at least one database compared to 30% of *P. laevis* transcripts matched with counterparts in a custom database^32^. This difference likely reflects the inclusion of *P. laevis* and other closely related species in the NCBI database following the earlier studies. However, only 14.6% of our transcriptome data matched the SwissProt database, while 29.3% matched entries in the PFAM or GO databases. This is due to the fact that PFAM and GO provide more comprehensive and domain-based annotations covering a broad range of proteins and functions, whereas SwissProt provides highly curated annotations that focus on a narrower set of well-characterized proteins and may exclude novel or less-characterized proteins in *D. truttae.*

The results of the GO annotation of *D. truttae* unigenes were consistent with those of the GO analysis of the *P. leavis* transcriptome^32^. Our findings support those of Mauer et al. (2020) who reported that a significant proportion of the acanthocephalan transcriptome is dedicated to metabolism - an adaptation likely linked to their parasitic lifestyle and reliance on nutrient uptake via their body surface (e.g. GO term “binding”)^32^. While the distribution of biological process (BP) subcategories were similar in *D. truttae* and *P. laevis*, the molecular function (MF) categories related to binding, catalytic activity and transporter activity were more abundant in *D. truttae*. These proportions were comparable to those observed in the rotifer *Brachionus koreanus*^32,34^, indicating possible shared physiological traits related to environmental adaptation.

The species distribution of the sequences annotated in the NR database was consistent with expectations, given the scarcity of genomic and transcriptomic data available for acanthocephalans. As anticipated, the highest sequence similarity was with *P. laevis*, the only acanthocephalan species for which both genome and transcriptome data are available^32^. Mauer et al. (2020) also identified similarities between sequences from Acanthocephala, Rotifera (especially Bdelloidea), and Platyhelminthes^32^. The resemblance to bdelloid rotifers likely reflects the phylogenetic relatedness of these taxa, while the similarity to trematodes may be due to shared adaptations to a parasitic lifestyle.

Given the parasitic nature of *D. truttae*, there was a high probability of contamination of the studied parasite with the host fish tissue. However, the low percentage of matched sequences confirmed that this contamination was negligible. Similarly, Mauer et al. (2020) reported that only 4% of the contigs of *P. laevis* matched sequences in the genome and transcriptome database of its fish host (*Cyprinus carpio* Linnaeus, 1758), confirming that host contamination was insignificant^32^. The results of the KOG classification of *D. truttae* unigenes were comparable to those of the nematode *Angiostrongylus cantonensis* and the cestode *Taenia pisiformis*^35,36^. Although “general function prediction only” was not the largest cluster in these two organisms, this is not surprising in *D. truttae* due to the limited amount of genomic and transcriptomic data available for acanthocephalans. The occurrence of KEGG Orthology terms in the analysis of *D. truttae* was similar to that of *Trichinella pseudospiralis* and *T. spiralis*, especially in the KEGG BRITE categories^37^. This suggests a comparable metabolic profile, reflecting the evolutionary and functional parallels between these parasitic species.

The high percentage (> 20%) of genes that could not be assigned to an orthogroup in the OrthoFinder analysis could be due to incorrectly assembled or annotated genes or poor species sampling^38,39^. However, this result is not unexpected, as we are working with a non-model organism whose genome has not yet been sequenced. As Acanthocephala is a large taxonomic group for which only a single genome has been published^32^, we considered that Acanthocephala species are phylogenetically distant from other well-studied taxa^40^, limiting the ability to refine species selection due to the scarcity of available comparative genomic data. Given that we used NCBI reference genome data for all species except *D. truttae*, we did not interpret unassigned genes as assembly errors, but instead chose to report the full set of results. The high number of potentially species-specific genes and the low number of single-copy orthogroups are consistent with the diversity and phylogenetic distance of the species included in the analysis^38,39^. However, the percentage of species-specific genes is notably higher than the values commonly reported in the literature^41,42^. For comparison, an analysis of the transcriptomes of larvae of the parasitic nematode *Anisakis pegreffii* revealed that 58.1% of all unigenes obtained by *de novo* assembly were novel and that only 41.9% corresponding to previously known genes. This finding supports the hypothesis that the high specificity may reflect the lack of comprehensive comparative transcriptomic data rather than methodological issues^43^.

The percentage of metal-binding proteins in a proteome can vary depending on the organism and the specific environment. However, it is generally estimated that 20–30% of all proteins in a typical proteome are metal-binding, with the majority of binding the essential metals, such as Ca^2+^ and Mg^2+^. Using MeBiPred software, our analysis predicted 14.5% of proteins binding the corresponding ions (Fe, Ca, Na, K, Mg, Mn, Cu, K, Co, and Ni), similar to another Acanthocephala *P. laevis* (15.5%). This percentage is slightly lower than that of the reference proteome of the alga *Micromonas pusilla* (19.4%), which was analyzed using the same method^30^. This lower number could be a consequence of the two-tiered approach of the software, since some proteins classified as members of the families that bind metals according to the Swissprot or PFAM databases were characterized by MeBiPred as non-metal-binding.

The predominance of zinc-binding proteins, which account for 53.8% of MBPs in *D. truttae* and 47.2% in *P. laevis*, emphasizes the essential role of zinc in various biological processes. Zinc is crucial for DNA replication, repair, transcription and protein synthesis and thus essential for cellular functions. Zinc fingers, a common motif, are particularly involved in gene regulation, which may explain its prevalence among the proteins in these organisms. The dominance of zinc finger proteins in *D. truttae* (246 sequences) is consistent with observations in other invertebrates and eukaryotic organisms such as *Caenorhabditis elegans* (Nematoda)^44^ and *Drosophila melanogaster* (Arthropoda)^45^, in which zinc fingers also represent a large proportion of the proteome, reflecting their essential role in regulating gene expression during development, differentiation, and stress response. Zinc finger motifs, abundant in these proteomes, may reflect the evolutionary pressure to maintain robust transcriptional control mechanisms in dynamic host environments.

Phylogenetic analysis of metalloproteases, another group of zinc-binding protein in *D. truttae*, shows that the sequences for reprolysin and matrixin do not form distinct clusters but are dispersed across different clusters. Very strong evolutionary conservation or functional similarity between the metalloproteases of *D. truttae* and *P. laevis* was expected, since they both belong to Acanthocephala. Rotifera, Nematoda and Hemichordata showed a slightly lower level of conservation, while Platyhelminthes showed a moderate range of low e-values in BLAST alignments, suggesting a somewhat reduced degree of conservation. Metalloproteases in Acanthocephala are predicted to degrade extracellular matrix (ECM) components, facilitating the tissue invasion, immune evasion, and nutrient acquisition. These processes are similarly observed in nematodes and parasitic platyhelminths, suggesting convergent evolution driven by parasitic lifestyles. However, the differences in sequence conservation among taxonomic groups (e.g., lower conservation in platyhelminths) may point to unique evolutionary adaptations in acanthocephalans^46,47^. The analyzes of MBPs defined by PFAM as iron-binding in *D. truttae* were focused on the iron-sulfur group. Iron-sulfur proteins are involved in electron transfer and enzyme catalysis. They are ubiquitous and are found in all domains of life. In Acanthocephala they may play a role in the metabolic processes and energy production. As parasites, Acanthocephala can be confronted with fluctuating and rather low oxygen levels in their host’s intestine, which requires a flexible metabolic adaptation. Their adaptations include: (1) a relatively low metabolic demand, so that diffusion is sufficient for gas exchange, and (2) the ability for anaerobic metabolism, which is useful in the low-oxygen environment of their hosts’ gut^48^. Acanthocephala may have adapted mitochondrial function through iron-sulfur proteins in complex I and complex II to flexibly switch between aerobic and anaerobic metabolic pathways. Acanthocephala also possesses machinery for the assembly of iron-sulfur clusters, as we found some of its components in the proteome of *D. truttae*, based on the definition of PFAM domains (frataxin or frataxin-like and ferredoxin or ferredoxin-like proteins). Due to the low e-values obtained from the BLAST alignments, the degree of concordance between the sequences of *D. truttae* and *P. laevis* was, as expected, the highest. Nematoda, Platyhelminthes, and Hemichordata also show very low e-values in most cases, suggesting conserved sequences, though with some slight variations. A few outliers with higher e-values (e.g. 0.001 for Nematoda, 0.028 for Platyhelminthes) may suggest some divergence and possible adaptations of acanthocephalan proteins compared to these taxonomic groups. Nickel-binding proteins, although less abundant, play a key role in the activity of certain enzymes such as urease and hydrogenases, which are involved in nitrogen metabolism and energy production^49^. The presence of nickel-binding proteins suggests that *D. truttae* requires nickel for certain metabolic functions, particularly those related to environmental adaptability. The analyzed sequences are highly conserved, particularly in Acanthocephala and Rotifera. The variations in e-values across taxonomic groups (e.g. in Nematoda) may suggest that these sequences have diverged functionally or evolutionarily from the Acanthocephalan sequences.

561 protein sequences that had no characteristic metal-binding domains based on the PFAM database, were grouped using MeBiPred software to predict their metal-binding preferences. This approach enabled categorization of proteins based on their predicted ability to bind specific metal ions. The hierarchical cluster map was used to visualize the patterns in metal-binding preferences, resulting in the identification of three main clusters (Fig. 9, Supplementary File 8). An important observation across all clusters is the almost equal preference for Zn ions. This likely reflects the importance of Zn as a ubiquitous metal cofactor involved in a variety of biological functions, such as enzyme catalysis, structural stabilization, and regulation of protein-DNA interactions^50^. Overall, the proteins in all clusters show a lower preference for binding Mg, Fe and Mn. This suggests that the remaining proteins are not as strongly involved in functions where these metals play critical roles, or that they are specialized in functions involving other transition metals, particularly Zn, Ni, Co, and Cu. The Zn and Cu binding preferences indicate functions in metalloenzymes, redox biology, or metal ion transport, while the Ca preference indicates calcium-dependent signalling or structural functions^16^. Proteins with low Fe binding preference may not be involved in iron-sulfur cluster formation or electron transport, but their high affinity for other metals suggests alternative biological pathways. These findings highlight the critical role of zinc-binding proteins in parasite survival, supporting enzyme catalysis, structural stabilization, and immune evasion. The low preference for iron-binding proteins suggests an adaptation to avoid host-imposed iron limitations, favouring zinc, copper, and nickel-dependent pathways instead. This may enhance parasite resilience against host defences and oxidative stress. Additionally, calcium-binding proteins indicate roles in signalling and motility, aiding host invasion. Environmentally, parasites may influence zinc and copper cycling in ecosystems, while their reduced reliance on iron and magnesium suggests minimal contribution to traditional iron-sulfur and electron transport processes. Overall, these adaptations reflect a strategic shift in metal utilization, impacting both host-parasite interactions and environmental metal cycling.

We have decided to narrow our analyses strictly to the several specific PFAM protein groups known to be involved in metal binding. The group with the largest number of proteins was PF00005, which includes ABC transporters. This was expected, as this group contains a large family of proteins responsible for the translocation of a variety of compounds across biological membranes. The finding that only 11.0–24.0% of the ABC transporters are predicted by MeBiPred to be metal-binding proteins suggests that although the “ABC transporter” family contains proteins that can transport metal ions, this is not the main function of most of them. *D. truttae* had an MBP from a member of the “metallothionein” family PF01439, which appears to be closest to the Class II metallothioneins, under PFAM number PF00131. The metallothionein-like protein type 2 (MT-2) is expressed in a wide range of organisms from different kingdoms, including animals, plants, fungi and even some bacteria. Various invertebrates, including mollusks and arthropods, express MT-2, which helps detoxify heavy metals accumulated from their environment^51,52^. Although proteins in the family "Heavy-metal-associated domain" are found in both microorganisms and mammals and transport heavy metals, they do not appear to play a role in Acanthocephala, as we did not find MBPs in *D. truttae* or *P. laevis*. Two members of the "ATP-binding cassette" protein group had metal-binding properties, and only one of them had the highest percentage of sequence similarity with the similar protein sequence of *P. laevis*, which in contrast was characterized as non-metal-binding. Whether this difference is a consequence of the two-tiered method used by the MeBiPred software to identify metal binding, or because the sequences were too different, remains to be clarified in future studies. Data availability is a major limitation in this study due to the lack of reference genomes and general molecular data on Acanthocephala in databases. In contrast to model organisms with extensive reference genomes and databases, there are no previously published genomic resources for *D. truttae*, making comparative analyses challenging. A limiting factor in our research on MBPs could also be the limited capacity of protein databases and software. The MeBiPred software has been trained and optimised based on well-characterised model organisms^28^. Its performance may decrease when applied to non-model organisms with unique or poorly studied molecular features. In addition, the PFAM database is more comprehensive for well-studied model organisms. For organisms that are not model organisms, such as Acanthocephala, the database may lack annotations for certain proteins or domains that are unique to these species. The PFAM approach is based on sequence homology and Hidden Markov Models (HMMs), which can miss highly divergent sequences or novel protein domains that are not captured by existing HMM profiles. The focus of the study on samples of *D. truttae* from the Krka River limits its scope, as genetic divergence between the Croatian population and the populations in Bosnia^53^ and Italy may exist, leading to a lack of generalisability to the entire range of the species. Regarding sample composition, while pooling helped minimize individual variability, it may have masked variations in MBP expression between individual parasites.

Future research on MBPs could explore comparative transcriptomics to reveal metal-binding gene expression, qRT-PCR and functional assays to validate MBP activities, and environmental sampling with lab exposure to study metal concentration effects. Single-cell proteomics may also uncover cell-specific MBP expression and low-abundance proteins.

## Conclusions

In this study, we present the first draft of the entire transcriptome, which is representative of adults of both sexes of the freshwater acanthocephalan *Dentitruncus truttae*. This is the first publicly available transcriptome of the family Leptorhynchoididae and serves as a valuable addition to the field of environmental parasitology The dataset, which yielded an average of 12.8 Gb of clean reads, was analyzed alongside the transcriptome and genome data of *Pomphorhynchus laevis*—the only acanthocephalan sequenced to date - and revealed a 47% sequence similarity highlighting conserved genomic regions within acanthocephalans. Orthology assessments across species of Rotifera, Nematoda, Platyhelminthes and Hemichordata emphasise the evolutionary proximity of *D. truttae* to *P. laevis*, followed by Rotifera and Nematoda. The comprehensive annotation of the transcriptome of *D. truttae* using seven databases reveals a strong enrichment of genes related to metabolic processes — a likely adaptation to its parasitic lifestyle characterised by nutrient uptake via the body surface. The transcriptome of *D. truttae* provides valuable insights into its role in environmental surveillance and host-parasite coevolution, particularly in salmonids. Due to the increasingly recognized importance of the ability of acanthocephalans to effectively accumulate toxic metals and influence the metal exposure of their hosts, this study also focused on metal-binding proteins (MBPs).

The analysis revealed numerous MBPs, particularly zinc-binding proteins, as well as others that interact with nickel, iron and copper. These proteins, which are associated with stress responses, detoxification and metal transport, can serve as biomarkers for metal exposure and bioaccumulation. By identifying genes that are upregulated in response to metal accumulation, diagnostic tests, such as qPCR panels, can track environmental contamination based on changes in *D. truttae* gene expression. As a freshwater species that bioaccumulates metals, *D. truttae* provides species-specific molecular indicators of ecosystem health, while comparative studies can reveal conserved metal-binding pathways for universal biomarkers in all aquatic systems. The transcriptome also sheds light on molecular mechanisms of metal tolerance, contributing to conservation and bioremediation strategies, such as recombinant protein production for metal sequestration or engineering bioindicator organisms. Insights into host-parasite interactions reveal genes related to immune modulation, nutrient uptake, and defense evasion, indicating that *D. truttae* thrives in metal-rich environments by imposing selective pressure on its host’s detoxification and immune responses. This could lead to a co-evolutionary “arms race” in which the genomes of host and parasite adapt to environmental stressors such as metal pollution. Genetic models built from transcriptomic data can simulate coevolutionary processes and predict how host-parasite dynamics evolve under environmental pressure. This research explains phenomena such as parasite resistance, host tolerance, and the evolutionary effects of metal stress on immune gene selection and deepens our understanding of the ecological and evolutionary consequences in aquatic ecosystems.

Overall, the transcriptomes of acanthocephalans seem to be streamlined and specialized for their parasitic lifestyle. They focus on genes necessary for survival and reproduction in their hosts, in contrast to free-living organisms, which have more complex and diverse transcriptomes, reflecting their adaptability to different environmental conditions and ecological niches. The results presented provide a valuable basis for further investigations of metal homeostasis in these parasites and for solving many questions about the phylogeny, taxonomy, diversity and evolution of Acanthocephala.

## Materials and methods

### Sampling

The adult parasites used for analysis in this study were collected from the intestines of brown trout caught in the Krka River in Croatia. The Krka River is a typical karst river that flows through the Dinaric ecoregion of the Western Balkans and discharges into the Adriatic Sea. Sampling was conducted in April 2021 at two sites in the upper reaches of the river: (1) the river source (KRS), known for its high water quality, and (2) approximately four kilometers downstream (KRK), near the Town of Knin (population: 8,300), which is impacted by industrial (screw factory) and municipal wastewater discharges (Fig. S8). Previous studies have indicated differences in metal accumulation and stress responses between fish from these two sites^54,55^.

Fish were collected by electrofishing in accordance with the Croatian standard protocol HRN EN 14,011 (2005)^56^. The fish were anesthetized and sacrificed using unbuffered tricaine methane sulfonate (MS 222, Sigma Aldrich) following the Ordinance on the protection of animals used for scientific purposes^57^ at a concentration of 50 mg/L, as recommended by Topić Popović et al. (2016) and Xu et al. (2008)^58,59^. To minimize individual variability, six acanthocephalan specimens were isolated from the intestine of each fish, rinsed in phosphate-buffered saline (pH 7.4), pooled, placed in cryotubes and frozen in liquid nitrogen. Upon return to the laboratory, these pooled samples were stored at -80 °C until further analysis. Each pooled sample was visually inspected to confirm the presence of both male and female individuals, ensuring balanced representation to avoid potential bias from sex differences during transcriptomic analysis and to maximize transcript coverage^32^.

### Total RNA extraction and RNA sample quality control

Workflow for the RNA-Seq analysis and Identification of MBPs in *D. truttae* is summarized in Fig. S9. Total RNA was extracted from the pooled samples (each containing six parasite specimens) using a Direct-zol™ RNA Miniprep Kit (Zymo Research) according to the manufacturer’s instructions. Prior to RNA isolation, the samples were homogenized using an Ultra Turrax T8 homogenizer (Ika Werke). RNA quantification was initially performed spectrophotometrically (BioSpec-nano, Shimadzu), and degradation or possible contamination was assessed by 1% agarose gel electrophoresis. After confirming their appropriate concentration and quality, samples were sent to Novogene (UK) Company Limited (https://www.novogene.com/us-en/) for commercial cDNA library preparation and RNA sequencing. Only high-quality total RNA (RIN > 7.5, without impurities) was used for further cDNA library construction and sequencing: five from the river source and three from the downstream anthropogenically impacted site.

### Library construction and sequencing

Libraries were prepared using the Illumina TruSeq RNA-Seq protocol, and sequencing was performed on an Illumina HiSeq 2500 platform, generating 150 bp paired-end reads with the TruSeq PE Cluster Kit v3 (Illumina). The raw data were stored in FASTQ format and quality of each RNA-Seq library was reviewed with FastQC software^60^. All the samples had a Q30 Phred score > 90% and a Q20 Phred score > 96% (Table S1). The raw reads were deposited in the Sequence Read Archive (SRA) database (NCBI accession number: PRJNA1123588). Clean reads were obtained by removing adapters, poly-N sequences, and reads containing more than 50% low-quality bases (Qphred ≤ 5) using the FASTQ Toolkit (v2.2.5 within BaseSpace).

### Transcriptome assembly and annotation

Trinity was used for *de novo* transcriptome assembly due to its effectiveness in reconstructing full-length transcripts and isoforms from RNA-seq data in the absence of a reference genome^61^. The assembly was performed using default parameters optimized for transcriptome data. Detailed information about the workflow is available at https://github.com/trinityrnaseq/trinityrnaseq. CORSET was used to reduce redundancies in the Trinity assembly by clustering transcripts based on shared mapping events and expression patterns, producing a non-redundant set of representative sequences^62^. Further details about the workflow are available at https://github.com/Oshlack/Corset/wiki. The completeness of the transcriptome assembly (Trinity.fasta, unigene.fasta, and cluster.fasta) was assessed using Benchmarking Universal Single-Copy Orthologs (BUSCO) software^63^ by searching each assembly for the presence of eukaryotic “core” genes, using the Metazoa database as the reference (database: metazoa_odb10).

To ensure reliable gene annotation and comprehensive coverage, from basic sequence alignment to advanced functional and pathway analysis, and a thorough understanding of gene function, a multi-database approach was used, comprising seven annotation databases, each with specific software and stringent parameters. These databases included the NT database (NCBI BLAST; threshold: 1e^− 5^); NR, SwissProt, and KOG databases (DIAMOND; threshold: 1e^− 5^)^64^; PFAM (HMMER; threshold: 0.01)^65^; GO (Blast2GO and Novogene scripts; threshold: 1e^− 6^)^66,67^; and KEGG database (KAAS; threshold: 1e^− 5^)^68–70^.

The use of 0.01 as a threshold for the e-value in the PFAM database was a reasonable compromise and balance between sensitivity and specificity^71,72^ to capture a broader range of potential matches^73^.

### Identification of orthologous sequences

Orthologous were identified using OrthoFinder (v2.5.5)^39^ by comparing the predicted coding sequences (CDS) of *D. truttae* with the genomic coding sequences of eight species available in the NCBI database: *Pomphorhynchus laevis* (GCA_012934845.2), *Adineta steineri* (GCA_905250115.1), *Rotaria socialis* (GCA_905331475.1), *Brachionus calyciflorus* (GCA_002922825.1), *Ancylostoma ceylanicum* (GCA_000688135.1), *Trichinella nativa* (GCA_001447565.2), *Dicrocoelium dendriticum* (GCA_944474145.2) and *Saccoglossus kowalevskii* (GCF_000003605.2). BLAST all-v-all sequence similarity searches were conducted using DIAMOND^64^. Orthologous genes were defined as pairs of genes descended from a single gene in the last common ancestor of two species, while orthogroups represented sets of genes descended from a single ancestral gene shared across multiple species^39^.

### Metal-binding protein identification

The investigation of metal-binding proteins (MBPs) in Acanthocephala species, such as *Dentitruncus truttae*, is essential for understanding the mechanisms underlying the bioaccumulation of metals in these parasites and their broader ecological roles. MBPs contribute to critical parasitic adaptation processes, including the degradation of host proteins and tissues (zinc-binding metalloproteases), respiration and energy production (iron-sulfur proteins), metabolic flexibility in nutrient-limited environments (nickel-binding ureases and hydrogenases), and protection against metal-induced oxidative stress (metallothioneins). These proteins also hold significant promise as biomarkers for environmental metal dynamics, offering insights into both host-parasite interactions and the impacts of metal contamination in aquatic ecosystems^16^.

To systematically study MBPs in *D. truttae*, several key bioinformatics and analytical steps were implemented. Firstly, the transcriptome of *D. truttae* was translated into amino acid sequences by extracting coding regions (CDS) from the assembled unigene sequences using the standard codon Table (5’ to 3’ orientation). The translated sequences were subjected to BLAST (Basic Local Alignment Search Tool) searches against the NR (Non-Redundant Protein) and Swissprot databases. The NR database was utilized for comprehensive sequence coverage, maximizing the likelihood of detecting homologous proteins across a broad range of species. The SwissProt database, known for its manually curated, high-quality annotations, was used to ensure accurate and reliable functional predictions, reducing the risk of erroneous annotations.

For unigenes that did not produce significant matches in the BLAST analysis, TransDecoder (v3.0.1) was employed to predict coding regions and determine the directionality of the sequences. This step was critical for refining annotations and identifying potentially novel coding sequences that might represent species-specific or less characterized MBPs. Once coding regions were confirmed, *Me*Bi*Pred*, a machine learning-based software specifically designed for predicting metal-binding proteins from sequence-derived features, was used to identify MBPs in *D. truttae*^28^. This tool follows a two-tiered approach, as described in Dedman et al. (2024), allowing for a robust and precise prediction of MBPs^30^.

The predicted MBPs were classified into homogeneous groups based on their binding specificity for zinc (zinc-binding metalloproteases), iron (iron-sulfur proteins), nickel (nickel-binding ureases and hydrogenases) and metallothioneins (Supplementary File 3, 4, 6 and 7).

The MBPs identified in *D. truttae* were compared to related proteins in *Pomphorhynchus laevis* using data from GenBank (NCBI accession GCA_012934845.2) to investigate potential evolutionary and functional differences between the two species. The protein sequences were aligned using MUSCLE 3.8.31 to ensure accurate sequence homology assessments. Phylogenetic analysis was performed using MEGA X with the Maximum Likelihood method and the Whelan Goldman + Freq model, generating phylogenetic trees to visualize evolutionary relationships.

To further assess sequence similarity and potential homologues, BLASTp searches were conducted against annotated proteins from taxonomic groups including Acanthocephala (taxid:10232), Rotifera (taxid:10190), Nematoda (taxid:6231), Platyhelminthes (taxid:6157) and Hemichordata (taxid:10219). This taxonomic range was chosen to detect homologous MBPs across closely and distantly related taxa, providing insights into the conservation and divergence of metal-binding functionalities.

The resulting e-values from BLASTp alignments were transformed with the negative logarithm (base 10) to enhance the visualization of sequence similarity. Heatmaps were generated using Python’s Seaborn library (the heatmap() function)^74^ to illustrate these patterns across multiple taxa, facilitating the identification of potential homologues and highlighting shared or unique MBPs. The heatmaps enabled the detection of conserved MBPs that may play universal roles in parasitic adaptation and revealed proteins with species-specific functions potentially linked to the environmental conditions faced by *D. truttae*. The next step was the functional characterisation of MBPs by identifying specific protein families with conserved domains associated with MBPs. For this purpose, we used the PFAM database due to its comprehensive coverage, robust statistical models (Hidden Markov Models, HMMs) and high-quality curated entries^75^. Protein sequences were scanned for conserved domains using PFAM seed alignments and the corresponding profile HMMs for different MBP families from the PFAM database. Key MBP families involved in metal homeostasis, transport and detoxification were selected to cover a range of biological functions important for parasite adaptation to metal-rich environments and included domains under the PFAM numbers: PF00005 and PF00664 (ABC transporters), PF01545 (cation efflux family), PF02535 (ZIP-zinc transporter), PF04145 (Ctr-copper transporter), PF00131 and PF07846 (metallothioneins), PF02798 (glutathione S-transferase), PF03770 (sco1 chaperones), PF12409 (P-type ATPase cation transporter), PF00210 (ferritin-like domains) and PF00403 (copper permeases). We used a strict e-value < 1e^-5^, which minimises the number of false-positive hits and ensures that only domain hits with high confidence associated with metal-binding functions are selected.

## Electronic supplementary material

Below is the link to the electronic supplementary material.

Supplementary Material 1

Supplementary Material 2

Supplementary Material 3

Supplementary Material 4

Supplementary Material 5

Supplementary Material 6

Supplementary Material 7

Supplementary Material 8

Supplementary Material 9

Supplementary Material 10

Supplementary Material 11

Supplementary Material 12

## Data Availability

Data is provided within the manuscript or supplementary information files. RNA sequencing data from the present study have been submitted to the NCBI Sequence Read Archive under accession number PRJNA1123588 and are available from June 1, 2025, at https://www.ncbi.nlm.nih.gov/sra/PRJNA1123588. Previously published datasets used in this study are available at NCBI: for *Pomphorhynchus laevis* at https://www.ncbi.nlm.nih.gov/datasets/genome/GCA_012934845.2/, for *Adineta steineri* at https://www.ncbi.nlm.nih.gov/datasets/genome/GCA_905250115.1/, for *Rotaria socialis* at https://www.ncbi.nlm.nih.gov/datasets/genome/GCA_905331475.1/, for *Brachionus calyciflorus* at https://www.ncbi.nlm.nih.gov/datasets/genome/GCA_002922825.1/, for *Brachionus plicatilis* at https://www.ncbi.nlm.nih.gov/datasets/genome/GCA_003710015.1/, for *Ancylostoma ceylanicum* at https://www.ncbi.nlm.nih.gov/datasets/genome/GCA_000688135.1/, for *Trichinella nativa* at https://www.ncbi.nlm.nih.gov/datasets/genome/GCA_001447565.2/, for *Trichinella patagoniensis* at https://trace.ncbi.nlm.nih.gov/Traces? run=SRR1971737, for *Dicrocoelium dendriticum* at https://www.ncbi.nlm.nih.gov/datasets/genome/GCA_944474145.2/, and for *Saccoglossus kowalevskii* at https://www.ncbi.nlm.nih.gov/datasets/genome/GCF_000003605.2/.
