# Supplementary material for: First insight into metal binding proteins from the de novo transcriptome of acanthocephalan parasite Dentitruncus truttae
Source: Sci Rep. 2025 Jul 18;15:26152. doi: 10.1038/s41598-025-11623-5 (PMC12274423; doi:10.1038/s41598-025-11623-5)
Supplement: Supplementary file 11 — Supplementary Material 11 [file 41598_2025_11623_MOESM11_ESM.docx]

**Table S1.** Summary of Sequencing Reads and Quality Metrics for all *Dentitruncus truttae* samples

| **Sample** | **Raw reads** | **Raw bases (Gb)** | **Clean reads** | **Clean bases (Gb)** | **Error rate (%)** | **Q20 (%)** | **Q30 (%)** | **GC content (%)** |
| --- | --- | --- | --- | --- | --- | --- | --- | --- |
| KRK1 | 45,150,536 | 13.55 | 44,404,752 | 13.32 | 0.03 | 96.44 | 90.69 | 45.30 |
| KRK2 | 46,762,347 | 14.03 | 46,077,088 | 13.82 | 0.03 | 96.56 | 91.15 | 45.25 |
| KRK3 | 42,313,970 | 12.69 | 41,651,417 | 12.50 | 0.03 | 96.24 | 90.56 | 44.87 |
| KRS1 | 42,871,002 | 12.86 | 42,313,716 | 12.69 | 0.03 | 96.11 | 90.34 | 45.06 |
| KRS2 | 40,360,276 | 12.11 | 39,785,263 | 11.94 | 0.03 | 97.09 | 92.29 | 44.85 |
| KRS3 | 43,244,708 | 12.97 | 42,616,956 | 12.79 | 0.03 | 96.25 | 90.58 | 45.30 |
| KRS4 | 42,530,934 | 12.76 | 41,915,758 | 12.57 | 0.03 | 96.33 | 90.78 | 45.86 |
| KRS5 | 44,124,102 | 13.24 | 43,419,812 | 13.03 | 0.03 | 96.53 | 91.19 | 45.06 |

Raw/clean bases – (number of raw/clean reads) * (sequence length); Gb – gigabases; Q20/30 - Phred values greater than 20/30 base number contain the percen tage of total base


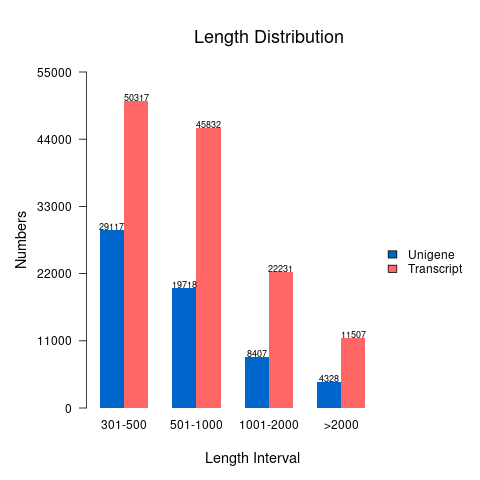


**Figure S1.** Length distribution of transcripts and unigenes sequenced from *Dentitruncus truttae*


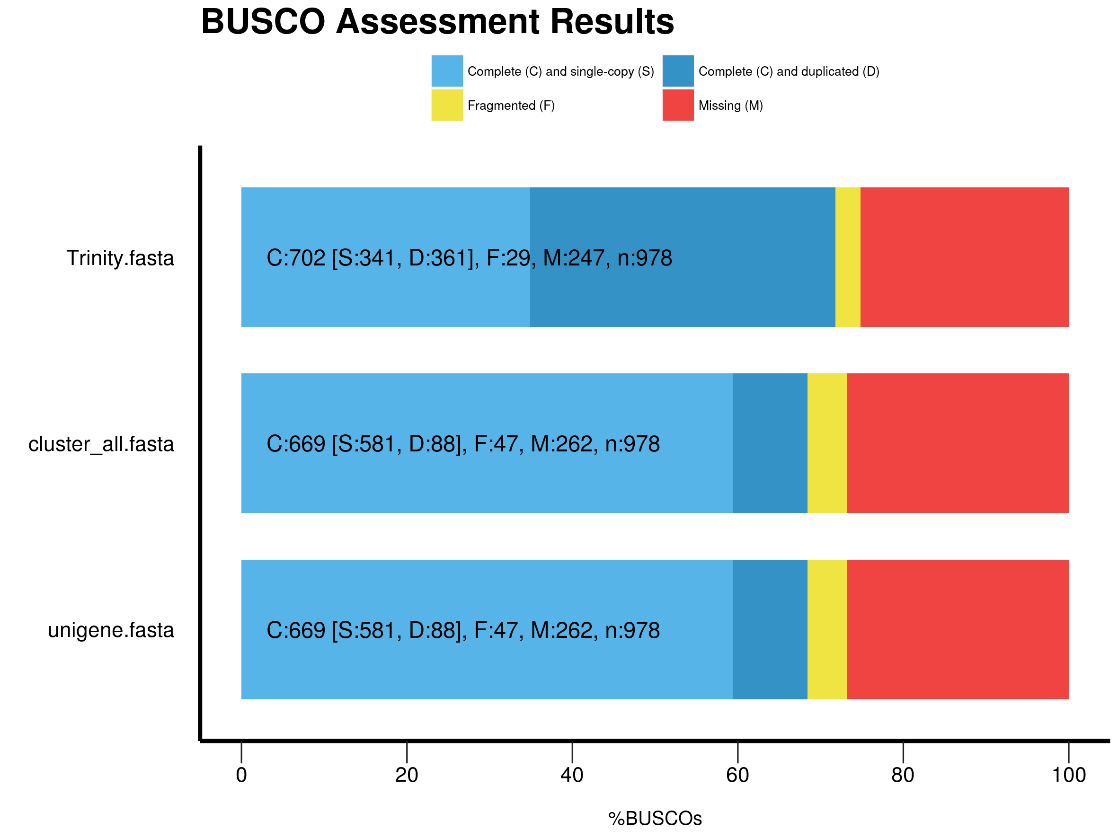


**Figure S2.** Evaluation result of the BUSCO analysis of the assembled transcripts


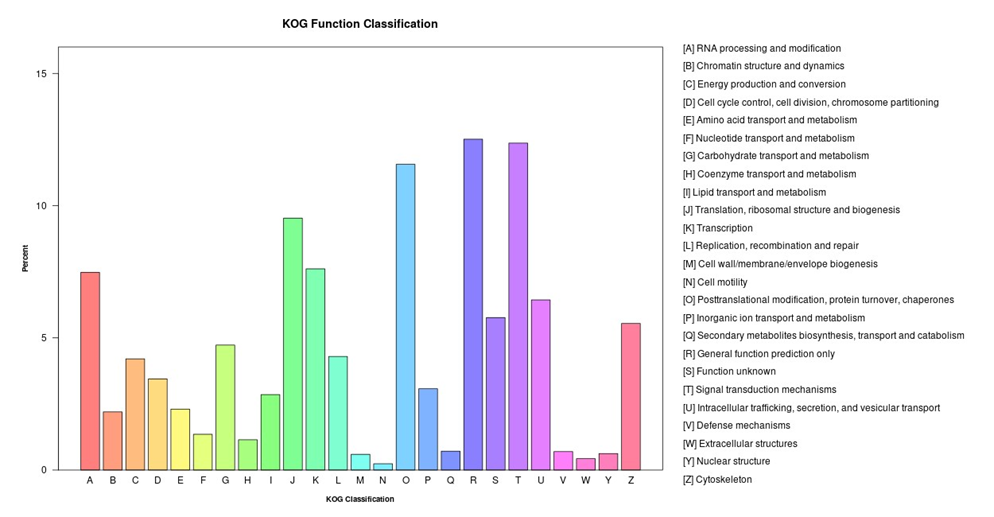


**Figure S3.** Histogram of the functional KOG classification of *D. truttae* unigenes

**Figure S4.** Percentage of *D. truttae* genes included in Brite hierarchies and different KO Pathway level 1 categories [68, 69, 70]

**Figure S5.** Distribution of gene annotations within selected Brite Hierarchy subcategories including: “Protein families: Genetic Information Processing” (e.g., Membrane trafficking, Chromosome and associated proteins, Messenger RNA biogenesis), “Protein families: Signaling and Cellular Processes” (e.g.,Exosome, Cytoskeleton proteins, Transporters), and “Protein families: Metabolism” (e.g., Peptidases and inhibitors, Protein phosphatases and associated proteins and Protein kinases) [68, 69, 70]


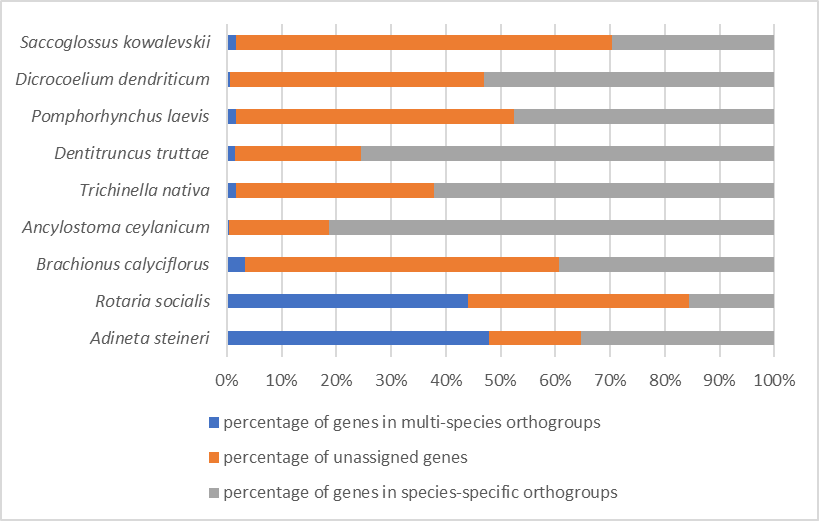


**Figure S6.** Distribution of genes across orthogroups in selected species by OrthoFinder

**
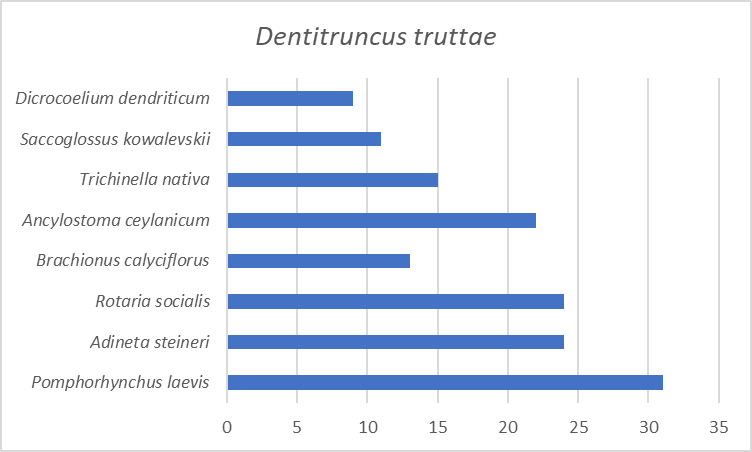
**

**Figure S7.** Number of shared orthogroups between *D. truttae* and other studied species


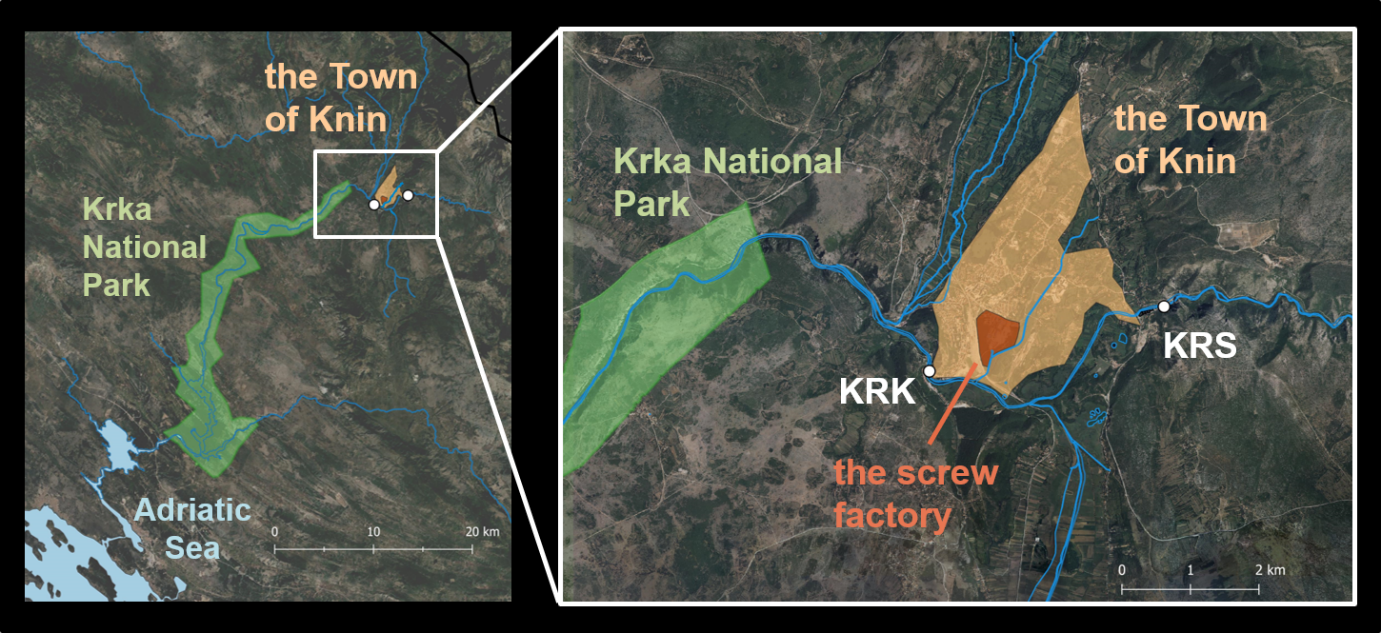


**Figure S8.** The Krka River with sampling sites at the spring (KRS) and downstream of wastewater discharges (KRK). The map was created in QGIS version 3.32 (<https://qgis.org/>) using the Digital Orthophoto Map DOF5 2019/2020 (available from the Croatian State Geodetic Administration Geoportal: <http://geoportal.nipp.hr/hr/nipp/pp/0969>) as the basemap.

**
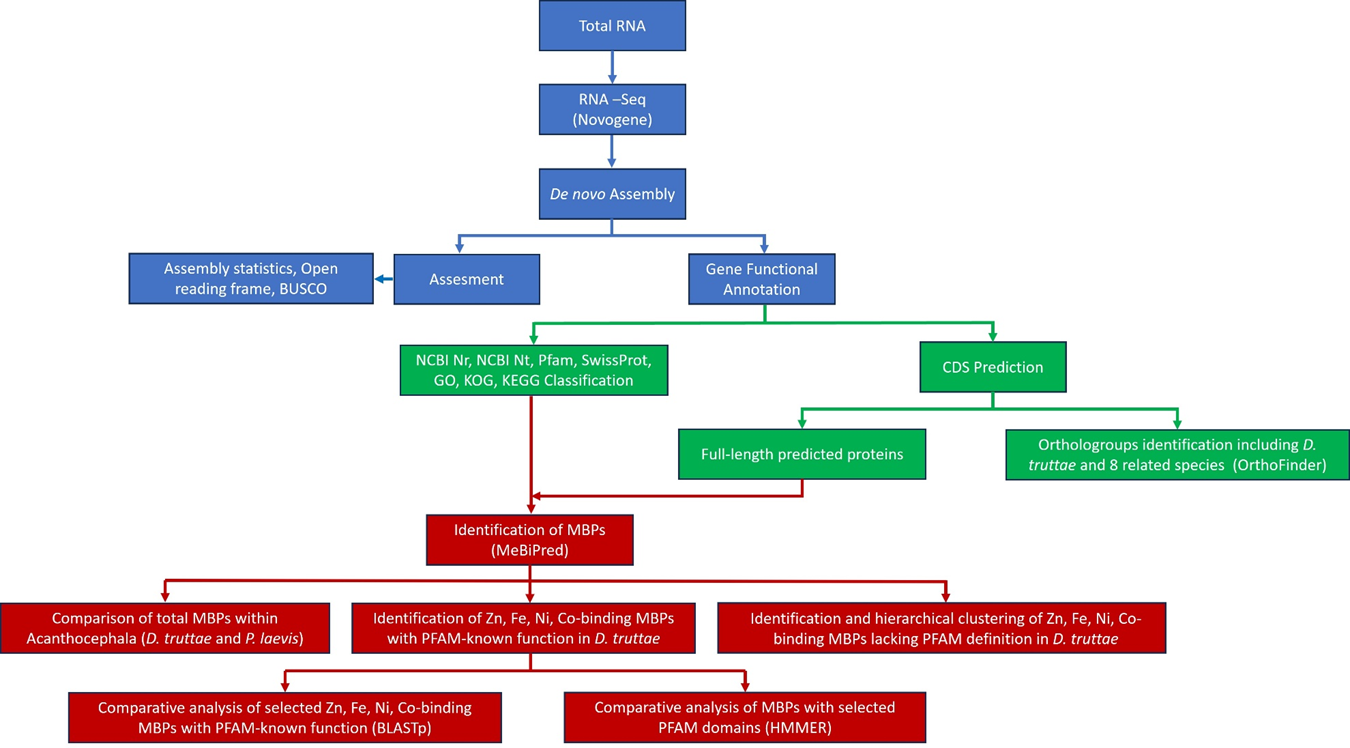
**

**Figure S9.** Workflow for RNA-Seq Analysis and Identification of Metal-Binding Proteins in *D. truttae*
