# Supplementary material for: First insight into metal binding proteins from the de novo transcriptome of acanthocephalan parasite Dentitruncus truttae
Source: Sci Rep. 2025 Jul 18;15:26152. doi: 10.1038/s41598-025-11623-5 (PMC12274423; doi:10.1038/s41598-025-11623-5)
Supplement: Supplementary file 12 — Supplementary Material 12 [file 41598_2025_11623_MOESM12_ESM.docx]

**Supplementary data**

**Supplementary File 2** –MeBiPred analysis of the transcriptome of *Dentitruncus truttae* and the coding regions of the *Pomphorhynchus laevis* genome: predicted metal-binding proteins and their metal-binding preferences.

**Supplementary file 2aMBP D truttae** - Amino acid sequences of MeBiPred-predicted metal-binding proteins from *Dentitruncus truttae*

**Supplementary file 2bMBP P laevis** - Amino acid sequences of MeBiPred-predicted metal-binding proteins from *Dentitruncus truttae*

**Supplementary File 3** - Metal binding prediction (MeBiPred) for *Dentitruncus truttae* zinc-finger proteins and metalloproteases

**Supplementary File 4** - Metal binding prediction (MeBiPred) for *Dentitruncus truttae* nickel ureases/hydrogenases

**Supplementary File 5** - Metal binding prediction (MeBiPred) for *Dentitruncus truttae* copper chaperones, copper oxidases and other copper-binding proteins

**Supplementary File 6** - Metal binding prediction (MeBiPred) for *Dentitruncus truttae* iron-sulfur group and other iron-binding proteins

**Supplementary File 7** – Characterization of metallothioneins identified in *Dentitruncus truttae* transcriptome

**Supplementary File 8** - Analysis of metal binding preferences using MeBiPred for metal-binding proteins lacking PFAM definition from *D. truttae*

**Supplementary File 9** - Metal binding prediction (MeBiPred) for *Dentitruncus truttae* specific Pfam groups
